# Optimized virtual reality-based *Method of Loci* memorization techniques through increased immersion and effective memory palace designs: a feasibility study

**DOI:** 10.1007/s10055-022-00700-z

**Published:** 2022-10-06

**Authors:** Brigham Moll, Ed Sykes

**Affiliations:** Sheridan College, 1430 Trafalgar Road, Ontario, ON Canada

**Keywords:** Virtual reality, Memory, Method of loci, Psychology, Cognitive science, Improving memory recall, Memorization

## Abstract

For most, an improvement in memory would always be desirable, whether from the point of view of an aging individual with declining memory, or from the perspective of someone seeking to memorize large amounts of information in the shortest period of time. One way for people to improve upon their memory performance is by using the *Method of Loci* (MoL), a famously complex, ancient memorization technique for non-spatial information recall. With the use of virtual reality technology, this technique can finally be easily taught to individuals for use in their daily lives. In this paper, we present an exploration into this avenue of using MoL in virtual reality and report on the design and evaluation of our new virtual memory palace that aims to prove the feasibility of improving upon designs from other studies to optimize memory recall performance. An experiment was conducted to evaluate our VR MoL environment. The results from week 1 on the pre-test (*M* = 62.55, *SD* = 24.01) and post-test (*M* = 82.91, *SD* = 15.99) memory task showed an increase in the number of words remembered was statistically significant, *t*(20) = -2.34, *p* = 0.014 where participants were able to remember approximately 20.4% more non-spatial information, when compared to traditional memorization techniques. After a second use, participants improved, remembering 22.2% more non-spatial information on the pre-test (*M* = 63.44, *SD* = 26.64) and post-test (*M* = 85.67, *SD* = 16.10) memory task, indicating that the increase in number of words remembered was statistically significant, *t*(16) = -2.142, *p* = 0.024. The results suggest that the virtual memory palace experience could be optimized to help participants learn the MoL technique with very little training time and potentially produce significant improvements in recall performance as a result.

## Introduction

In this paper, we present the results from an investigation involving the *Method of Loci* (MoL) memorization technique in an immersive Virtual Reality (VR) setting. *Loci* is the plural form of the Latin word for *locus*, which means *place* (Yates 1999).

**Fig. 1:**
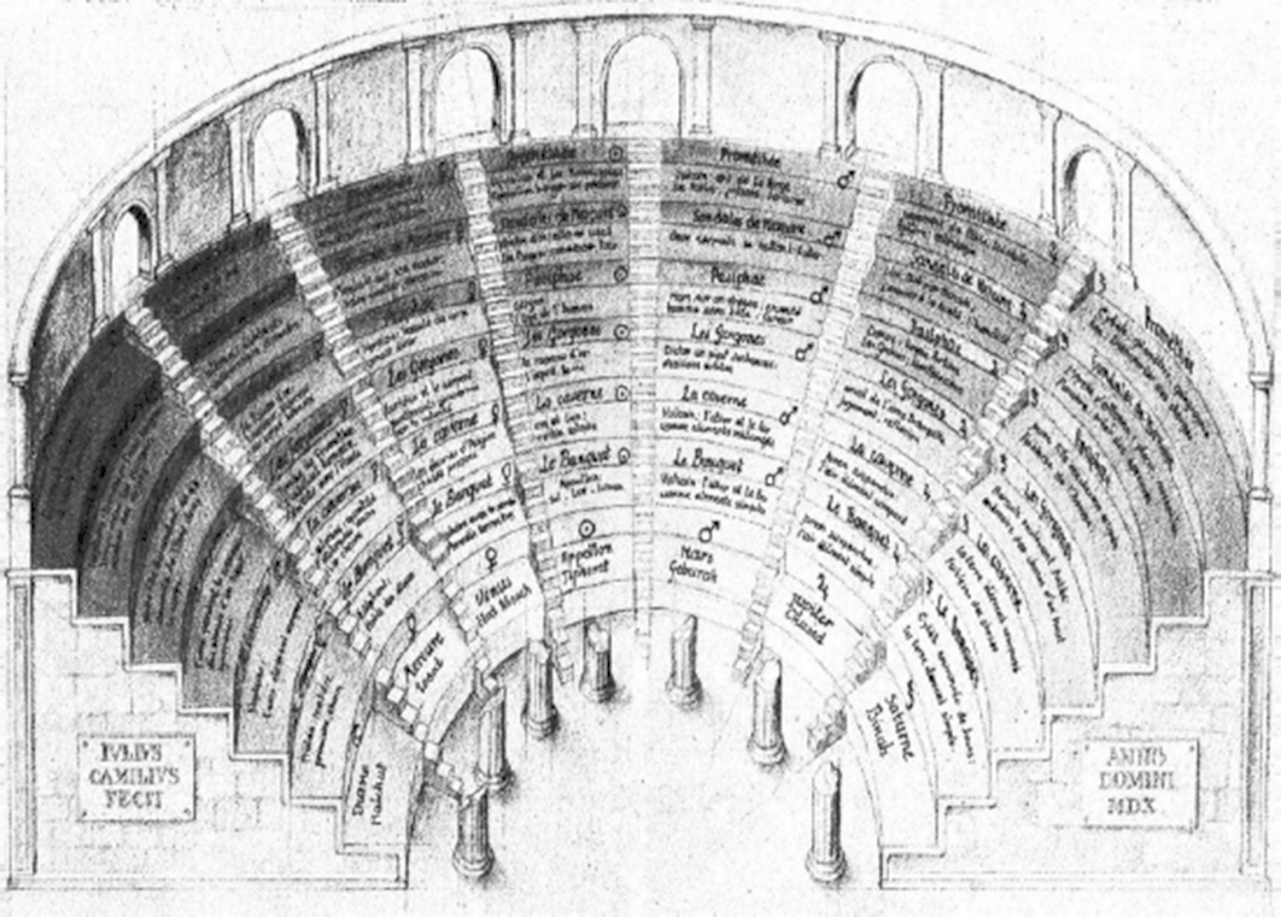
Depiction of a Memory Palace from 1511 AD, by Giulio Camillo Krokos et al. (2019)

The MoL is an ancient memorization technique known by many to be one of the most effective ways available for people to memorize non-spatial information by making use of the powerful spatial memory abilities exhibited by humans (Yates 1999). It was used by ancient Greek and Roman orators to remember lengthy portions of text. A sample visualization of the technique is shown in Fig. 1. Usage of the technique dates as far back in time as ancient myths such as in the story of Simonides of Ceos in the 5th century (Yates 1999).

In that myth, Simonides uses the MoL to remember the faces of various recently deceased people by imagining each of their seats at a table of a banquet hall. The technique makes use of the ability for a human to remember things based on places they have visited, and things they have seen, by asking the user of the technique to imagine a building in their mind, like a palace or a place from their past. Then, they are told to ‘place’ the different things they need to remember throughout this virtual building. Using a path defined by the memorizer through the imagined structure, the participant is able to remember everything s/he wanted to by simply finding ‘where’ the memories were placed.

A significant number of studies have been conducted developing this traditional mnemonic technique into a virtual or augmented reality (VR or AR) experience (Fettke et al. 2019; Huttner et al. 2015; Liu et al. 2019; Huttner et al. 2019; Krokos et al. 2019; Huttner and Robra-Bissantz 2017; Peeters and Segundo-Ortin 2019; Huttner et al. 2018; Vindenes et al. 2018; Bhandari 2019). In several papers, it was shown that people remember things more effectively with the traditional MoL. Research has shown VR enhancing the memorization process has significant potential, but more research is needed in this area (Krokos et al. 2019; Reggente et al. 2019; Peeters and Segundo-Ortin 2019; Huttner et al. 2019; Huttner and Robra-Bissantz 2017). The use of Virtual Reality MoL has already been shown to encourage people and increase people’s confidence in using the technique (Peeters and Segundo-Ortin 2019; Perera et al. 2019; O’Grady and Yildirim 2019; Huttner et al. 2015).

In this research, these ideas of exploiting VR and MoL are taken a step further. Past studies have not, for the most part, given participants full immersion into the MoL experience, so there was an aim to heighten immersion as much as possible in this work. This increased immersion is said to strengthen memory recall (Huttner et al. 2019; Krokos et al. 2019; Huttner and Robra-Bissantz 2017). By giving participants the opportunity to reach out and place objects with their actual arms, walk using their own legs, hear sounds in the virtual world and be fully immersed in the virtual experience, we postulate that this will have a positive impact on the effectiveness of the MoL such that it will result in better memory recall when memorizing non-spatial information.

While the traditional MoL has been shown to increase memory recall performance for quite some time (Yates 1999), it has always been considered complex and requiring much training for one to learn and use the technique effectively (Mccabe 2015; Huttner et al. 2015; Huttner and Robra-Bissantz 2017; Reggente et al. 2019; Legge et al. 2012; Huttner et al. 2019). Many attempts have been made with and without Head Mounted Displays (HMDs) to try and virtually simulate the memory palace of the MoL in order to lessen cognitive load of memorizers and make the technique more encouraging and accessible to all people. It is however an outstanding question in the field of cognitive science, memorization, memory recall and VR as to whether a virtual MoL technique can be impactful enough on recall performance in a short enough time for people to consider seriously using the MoL in their daily lives (Huttner et al. 2015; Bhandari 2019; Huttner et al. 2019; Putnam 2015). Increasing the use of MoL in VR would have some significant benefits such as mitigating memory issues caused by old age or disorders affecting cognition (Jjaz et al. 2019; Manivannan et al. 2019; Tuena et al. 2019; Sayma et al. 2019; Wiederhold and Riva 2019).

### Research goal

In this work, we designed a virtual memory palace for maximum memory recall performance based on suggestions from multiple other studies, engaged 11 participants, and evaluated the effectiveness of our system. This study involved an optimized VR MoL environment developed with heightened immersion when compared to other VR MoL systems such as those in Huttner et al. (2015), Vindenes et al. (2018), Huttner et al. (2018), Liu et al. (2019), Huttner et al. (2019), and Krokos et al. (2019) via an Oculus Rift S with the addition of sensors on the ankles and waist to simulate real walking movements (i.e., KAT VR’s ‘KAT Loco’ sensors). The system created allowed experimental information to be collected regarding memory performance of the participants under differing sensory stimuli aimed to increase immersion and thus strengthen memories in the virtual memory palace. Due to limited participants, this study is to be considered a feasibility study that has the goal of showing that optimization of the MoL through VR to allow participants to effectively use the famously complex memorization technique is in fact possible in a small amount of training time. The study hypothesis (*H*_0_) is: ‘Participants undergoing VR MoL will demonstrate better memory recall than traditional memorization techniques, and will be able to learn the MoL technique within a limited training time.’

## Related work

This section presents the relevant work in the context of proposed research: Traditional MoL; Immersion Supporting Recall in VR & MoL; VR Immersion; and VR Memory Palace Design Considerations.

### Traditional MoL

The Method of Loci technique involves picturing one’s self in a place (also known as the memory palace) that is known particularly well by the memorizer. Once prepared, knowing what they want to memorize, they imagine moving through the place in a particular path, placing things they wish to remember in various places throughout the palace. After the exercise, when they wish to recall the information, the memorizer merely recalls the place and walks through it the same way again in their mind, looking to the places they placed memories to remember each of them.

### Immersion supporting recall in VR & MoL

VR and the MoL have been shown to be useful in multiple studies for helping people recover from injuries or losses, and overcome mental disabilities or deficiencies (Tuena et al. 2019; Sayma et al. 2019; Manivannan et al. 2019; Wiederhold and Riva 2019; Jjaz et al. 2019; Optale et al. 2010). In a study on using VR environments for neurorehabilitation (Tuena et al. 2019), results seemed to point toward the conclusion that the more immersive an environment is, the better the environment is for spatial memory improvement training. Sayma et al. in Sayma et al. (2019) also supports this idea on the benefits of immersion.

The idea is further supported in a study on a VR memory testing tool for seniors (Jjaz et al. 2019), where results showed immersion leading to better memory recall. Optale et al. in Optale et al. (2010) had a similar experiment that led to similar observed outcomes.

In the earliest example reviewed (Huttner and Robra-Bissantz 2017), recall results leaned toward VR via HMD being more effective than through a monitor display (Huttner and Robra-Bissantz 2017).

In Krokos et al. (2019), virtual MoL recall performance is compared between participants using a monitor display and participants using an HMD. Through an experiment that asked participants to memorize the faces and names of people while only having the ability to look around and not move in the virtual environment, results were found to show that even a small amount of immersion could improve upon memory recall. The literature review included in the study also supported this conclusion.

Immersiveness is again praised as a factor for improved memory recall in Huttner et al. (2018), where people are found to recall information better in virtual MoL when items to be remembered include not only the text of a word to be remembered, but an image too, following the idea of dual coding theory. It was recommended that future research should delve into animating items to be placed, trying to use audio cues and videos, or any other types of media, to see how they affect memory recall performance.

In Huttner et al. (2019), authors also come to the conclusion that high immersiveness in environments leads to better recall when using VR MoL after reviewing other research. It is said here that a minimum of 40 words seems to be a good number for showing the true potential of the MoL technique. There is also support from this research for the idea that longer training times are needed for virtual MoL, highlighting the importance of the environment being familiar to participants.

### VR immersion

Examining past research in VR MoL experiments, there is a significant number of researchers encouraging increased immersion for better recall of information (Huttner et al. 2019; Krokos et al. 2019; Huttner and Robra-Bissantz 2017). Further review was done on two papers that investigated how to increase immersion in simulated environments (Sanchez-Vives and Slater 2005; Kong et al. 2017). Both studies expressed through experimental results and review of other research that virtual body representation is important. Not being able to see one’s self in a simulated environment can be strange to people, and it is recommended that a virtual representation be synced with the movements of a participant (Sanchez-Vives and Slater 2005; Kong et al. 2017).

When able to see one’s own hands or legs moving where they expect them to, and at the times they are expected to move, people feel a higher sense of ownership and agency over their virtual bodies (Kong et al. 2017). This leads to people feeling more like they are truly in the environment as if it were real, and theoretically this should lead to better memory recall in a VR MoL scenario.

Other studies, such as Sanchez-Vives and Slater (2005), supported 3D sound as an important factor to immersion. Haptic feedback when touching virtual surfaces, realistic walking, meaningful movements of the actual body of a participant, and inducing any intense emotion like fear are also mentioned by the authors as ways to improve upon making the participant feel more like they are present in a virtual environment.

### VR memory palace design considerations

In Liu et al. (2019), a pilot experiment is described where people can walk naturally unlike most research done before, using an allotted space in real life that matches the size of the rooms in the virtual memory palace. They could also reach out with controllers in each hand to interact with the environment, but items to be remembered were pre-placed throughout, so this interaction was seen by participants to be useless. Rooms were all identical in size and there was no walking transition between rooms, so the design of the memory palace was found to be confusing by participants. Traditionally, the MoL has been seen to work best with unique environments that are non-repetitive, and ones with an abundance of space between items to be remembered (Yates 1999).

**Fig. 2:**
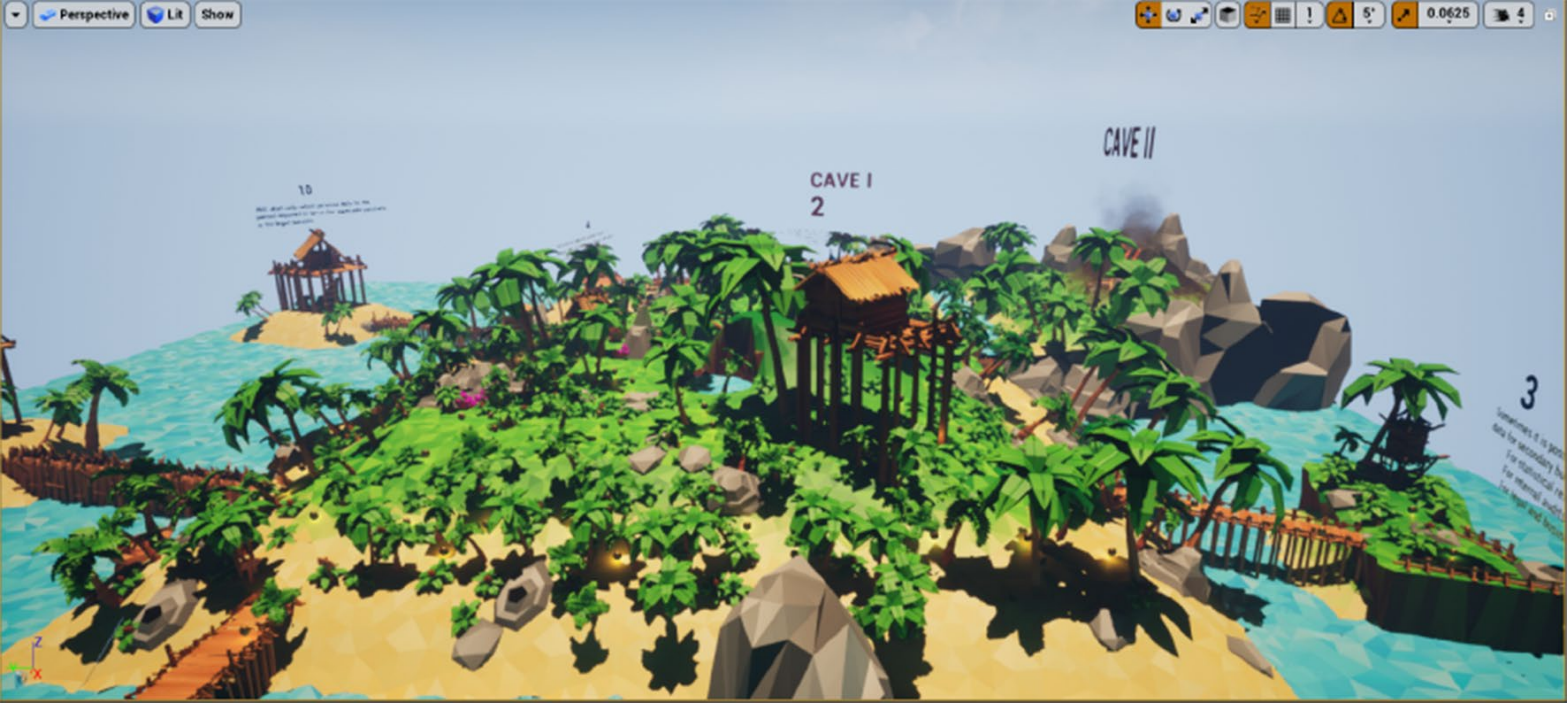
Environment from Bhandari (2019) inspired by the MoL

In Bhandari (2019), an experiment involving an environment for participants to explore based upon the concepts of the MoL showed high compliance to use the method, supporting the idea that these VR MoL environments are quite encouraging compared to traditional methods of learning (Bhandari 2019). Participants were given paths of coins to collect for exploration of the area, and asked to memorize large amounts of floating text. There was also a 3D sound experience to further the immersive experience (Bhandari 2019). This simulated environment is shown in Fig. 2.

A study (Peeters and Segundo-Ortin 2019) that reviewed various other VR MoL studies as well as more traditional MoL, investigated why the MoL is so effective, and how they might come up with design advice for future VR MoL experiments where high memory recall performance is the goal. The authors suggest increasing immersion as much as possible, lighting up potential areas of interest or using unique landmarks for placing things to be remembered in the palace, making participants use physical body movements to interact and place memorized things as well as move about, and make it so that participants can choose or even better, create imagery for items to be remembered when tasked with memorizing them. They also express interest in future studies of animating things to be remembered through moving objects or videos.

## Methodology

This section describes various aspects of the methodology including: (i) Design of the Memory Palace; (ii) Materials; (iii) Participants; (iv) Overview of the Experiment Design; (v) High-level Architecture of the VR MoL Environment; (vi) Pilot Study; (vii) Main Experiment; (viii) Internal and External Validity; and (ix) Analysis techniques.

### Design of the memory palace

**Fig. 3:**
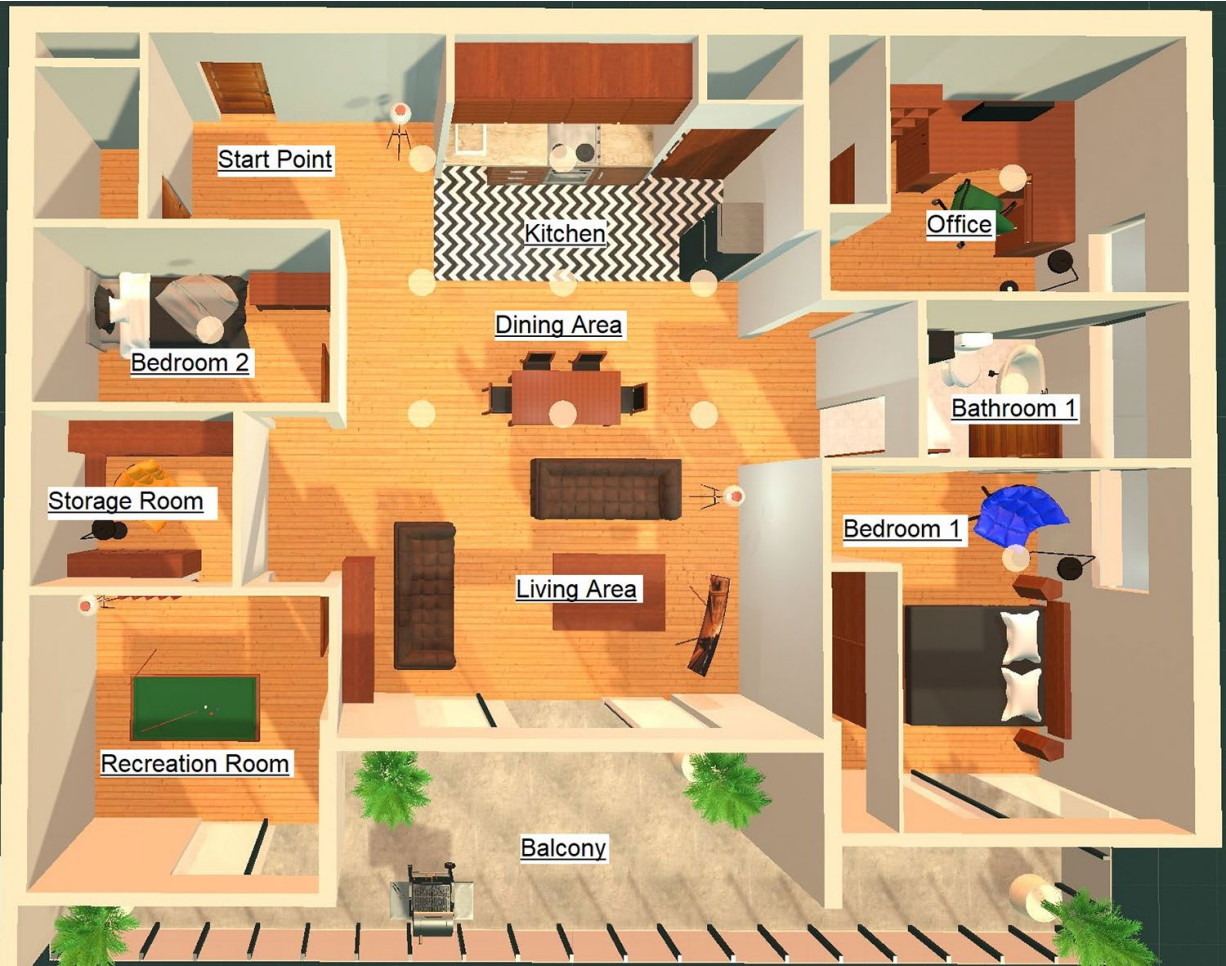
Virtual Memory Palace Apartment Design

**Fig. 4:**
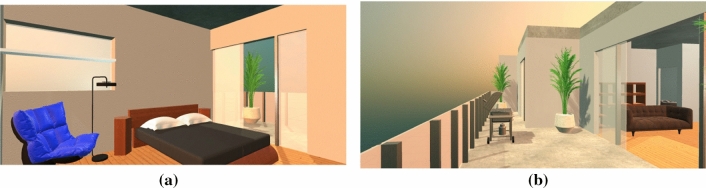
**a** Bedroom of the Virtual Memory Palace Apartment; and **b** Balcony of the Virtual Memory Palace Apartment

The simulated environment was designed according to the floor plan as shown in Fig. 3. An apartment was chosen for the palace because familiarity with the structure of an environment has been shown to produce slightly better memory recall results as opposed to other memory palace structure designs (Caplan et al. 2019). Also, multiple other studies have made use of palaces designed based on the structure of an apartment (Caplan et al. 2019; Huttner et al. 2019; Huttner and Robra-Bissantz 2017; Huttner et al. 2018, 2015; Legge et al. 2012; Jund et al. 2016). The 3D model of the apartment was visually designed and generated by Tyson M., an architecture degree graduate. The furniture models populating the apartment are free use license models used for purely academic purposes, made by other creators. The memory palace contains a kitchen, dining area, office, bathroom, two bedrooms, a storage room, a living area, a recreation room, and a balcony. Figure 4 presents two rooms in the memory palace. While an apartment’s general structure is familiar, it has also been said traditionally that the MoL should include usage of non-repetitive, distinct environments for more effective use of the mnemonic technique (Yates 1999). The apartment design was inspired by multiple high-end condo apartment layouts with the goal of creating an environment that looked distinct while not taking away from the idea of having a familiar, building-like structure appearance (Caplan et al. 2019).

### Materials

We created the VR MoL memory palace system using Unity 2019.2 and C#. The Oculus Rift S was used extensively for development, but our system can be generalized to most VR headset technologies (e.g., HTC Vive, etc.). The controllers associated with the headsets were integrated into the application using the Unity XR plugin management system. All visualizations from the VR environment were able to be viewed on a monitor running the Unity editor, where the researcher conducting the experiment was able to observe participants and record notes. Participants used the two touch controllers that accompany the Oculus Rift S for more realistic use of their hands and arms, and they also used KAT VR’s KAT Loco sensors to simulate realistic walking. The sensor devices have an open-source Unity SDK for integrating realistic movement into software for added immersion. Lastly, the cable coming out of the HMD was hung from the ceiling to be out of the way of participants using Hyperkin’s Freestep VR Cable Management System.

The KAT VR’s Loco sensors attach to the participant’s ankles and waist and are calibrated to ensure direct translation to the VR environment to interpret walking movements in as seamless and natural way possible. We incorporated these sensors into our VR environment because there is ample evidence that suggest that by incorporating natural human movement (i.e., physical walking) into the VR environment has shown to be an effective way to mitigate VR cybersickness (e.g., nausea, headaches and disorientation) (Corriveau Lecavalier et al. 2018).

### Participants

Due to the restrictions brought with the onset of COVID-19, convenience sampling was used to recruit participants in this study. Only those that could be allowed to be in close proximity of those running the experiment were permitted to be included in the participant pool. The study was conducted under an approved Research Ethics Board protocol at the academic institution (REB Protocol #: 2018-12-001-035). Furthermore, due to safety reasons, participants with conditions such as epilepsy were excluded. The researcher was present and in close proximity to the participant at all stages of the experiment to ensure their safety and comfort. In the end, we were able to engage 11 participants (min. age: 14, max. age: 65, mean age: 35.9, std. dev. σ: 14.9; eight males, three females; all adults had a post-secondary degree [i.e., college or university]). Each participant was told that the experiment would involve them using a virtual reality system in combination with an ancient memorization technique to memorize large amounts of information, and that their results would be compared against their typical studying strategies.

### Overview of the experiment design

This section presents an overview of the experiment design. We used a quasi-experiment design with repeated measures using one group pre-tests and post-tests. Participants were exposed to two treatments, each involving a pre-test and post-test, using the Method of Loci in our VR memory palace environment.

The experiment was conducted twice, on the same day of two consecutive weeks. This was done to see if there were any improvements in participant memory recall performance from the first attempt at the experiment compared to the second, when participants would be more familiar with using the VR MoL environment involved. Each week had entirely different word lists selected from the same overall pool of words discussed below. A few participants could not participate in the second week of the experiment due to conflicts in their schedules. We conducted the pre-test and post-test of this experiment on the same day, for each instance of the experiment in weeks 1 and 2. It is possible that due to this there may be some interference from the prior condition in the post-test. We hoped however that having completely separate word lists between the pre-test and post-test conditions would help to avoid this risk of interference.

Our experiment measured memory recall on words that had high imageability based on Madan et al. (2010). High imageability (or concreteness) is present in words that are easy for one to imagine or visualize in their mind (Legge et al. 2012). In Legge et al. (2012), using this same word pool, high imageability words were shown to lead to better memory recall performance in a virtual memory palace MoL scenario, as opposed to words of low imageability, and this encouraged our usage of the same word pool. For both the pre-test and post-test phases for our experiment, the words were randomly selected from a total pool of 108 words (Madan et al. 2010). Sample word lists for pre-tests and post-tests are shown in Table 1.

**Table 1:** Sample of randomly selected word list for the experiment (pre-test and post-test)

Sample pre-test word list			Sample post-test word list		
Bolt	Roast	Ankle	Canal	Feast	Gate
Crowd	Burial	Riot	Helmet	Rose	Hammer
Drive	Stable	Beard	Chapel	Cave	Limb
Twin	Toilet	Tank	Bishop	Troops	Museum
Cigar	Scarf	Sponge	Cart	Dwarf	Barrel
Basket	Lace	Tail	Onion	Flock	Blouse
Limp	Flame	Rubber	Drum	Deer	Infant
Veil	Bill	Meal	Salt	Tongue	Button
Stain	Card	Crest	Autumn	Disc	Wound
Beam	Bone	Devil	Essay	Cherry	Ladies

**Fig. 5:**
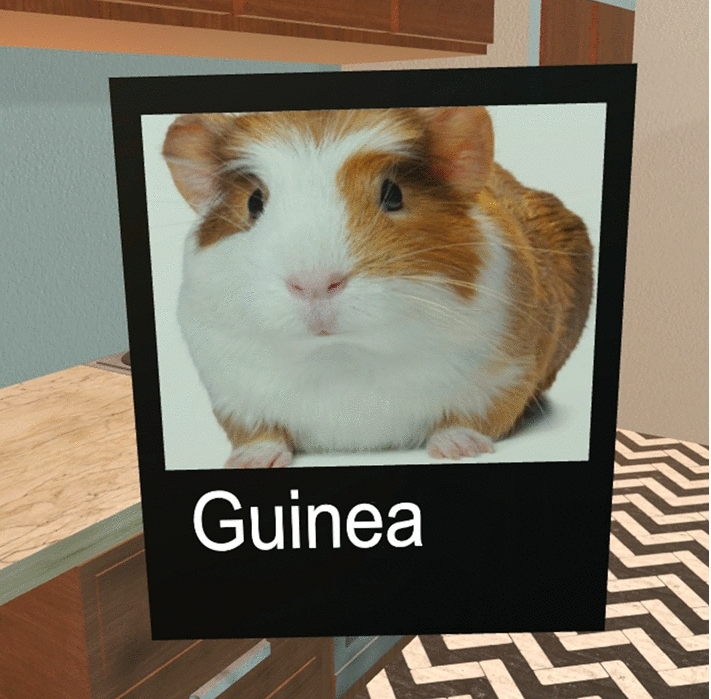
A *Placeable* object that represents a word on a word list to be remembered by a participant

For the pre-test, a list of words was given to each participant to memorize within 15 minutes before they were asked to recall them. Each participant then entered the VR memory palace and were given some time to become acquainted with the virtual environment. Participants were then asked to place 30 images (i.e., see Fig. 5) in memorable locations of their choice while attempting to implement the Method of Loci as explained to them in a traditional manner prior. After exiting the VR environment, participants were then asked to recall the words studied in the virtual environment.

### High-level architecture of the VR MoL environment

**Fig. 6:**
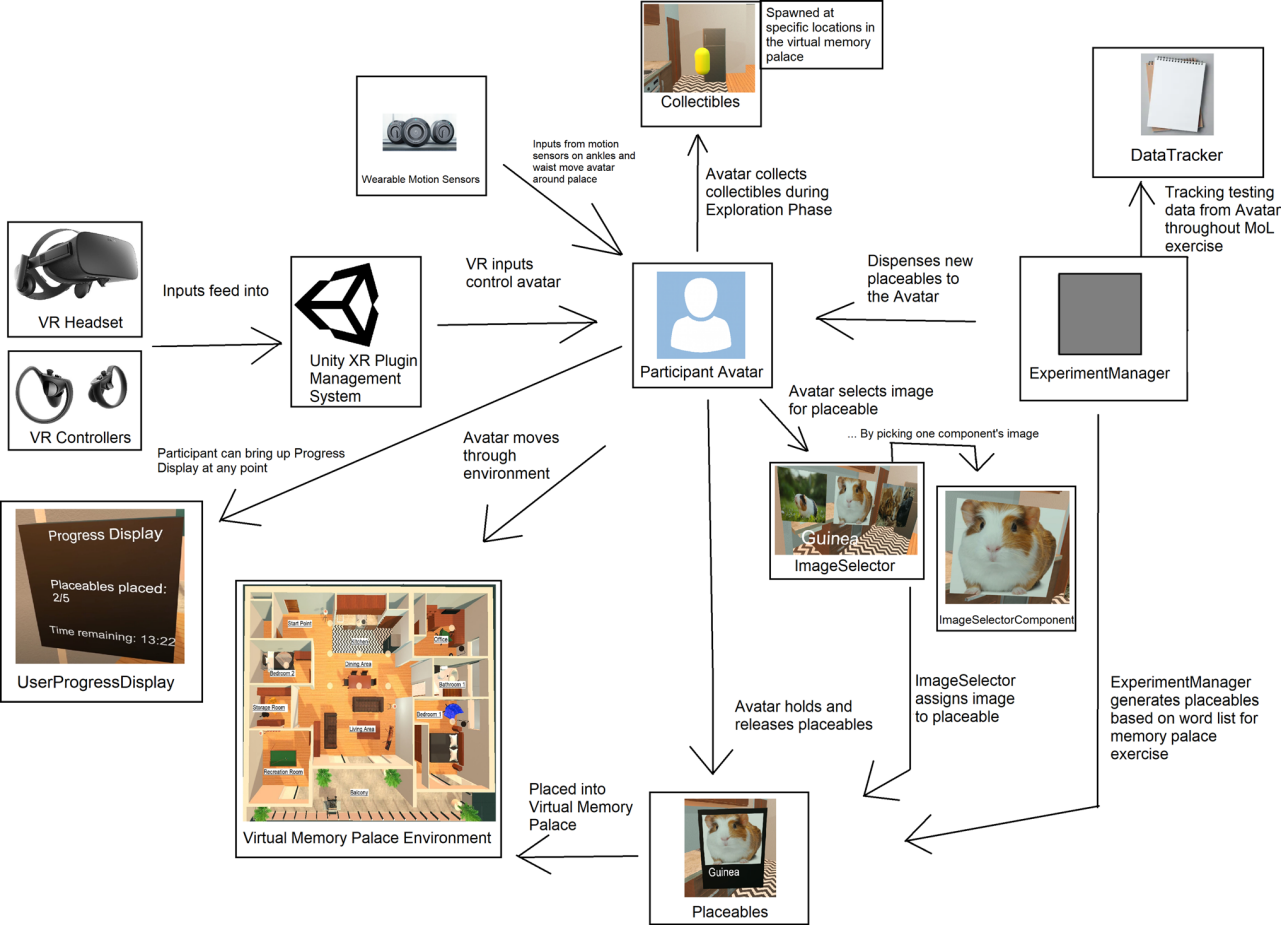
High-level architecture of the VR MoL environment

Figure 6 presents the high-level architectural model of the VR software system that we created to support this research. Unity’s XR management system and Oculus’ Integration package are used to integrate HMDs into the system. A Participant Avatar is made up of two gloved hands, representing the participant and controlled entirely by the inputs from the VR headset, controllers, and the worn ankle and waist sensors used for realistic walking.

**Fig. 7:**
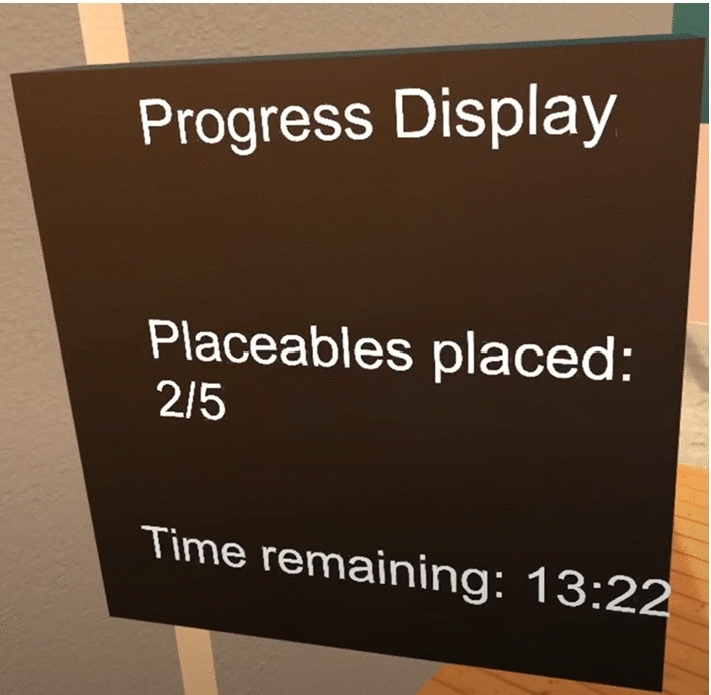
A *UserProgressDisplay* showing timing and progress information to a participant

Spawning of participant avatars to begin the experiment are controlled with an *ExperimentManager* object. This system keeps track of time limits in the Participant Training Phase and the VR MoL Phase. The *ExperimentManager* also tracks progress of collecting *collectible* objects in the area during the Participant Training Phase. For the Training Phase, key locations in the palace were chosen for collectible floating objects (*Collectibles*) to be spawned (*CollectibleSpawnPoints*). These objects disappeared when the participant touched them with either of their hands. The *ExperimentManager* collected metrics such as the number of collectibles remaining to be collected, and the number and location of placed objects to be remembered in the VR MoL Phase. This time and progress information was visible to participants through pop-up dismissible displays known as *UserProgressDisplays* that could be presented and dismissed with a simple button press on a controller. A *UserProgressDisplay* is shown in Fig. 7. The *DataTracker* records the time required by participants to place each object or collect each *collectible*, the locations of the objects placed, which images for each object were selected, and the order in which the *collectibles* were collected.

**Fig. 8:**
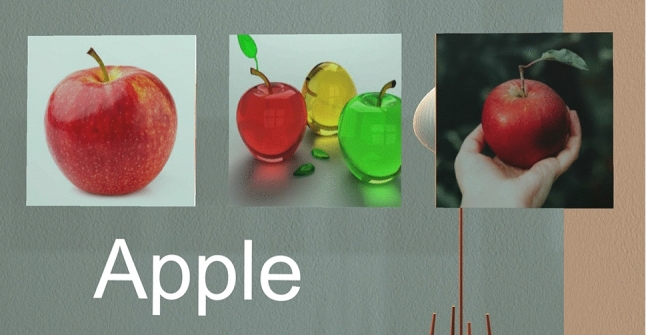
*ImageSelector* with three *ImageSelectorComponent* options for the word *Apple*

An *ImageSelector* provided participants with the ability to select an image from three presented (see Fig. 8) to them. This enabled the participant in the VR environment to have choice and presumably select the image that would be most personally memorable for them during usage of the MoL mnemonic technique.

Depending on which specific word was being placed next, three relevant images were presented in front of participants, and using their hands, they could reach out and touch one of the pictures to select it for the item to be placed. *ImageSelectors* spawned based on wherever participants were facing, with some distance in front of them so that each *ImageSelectorComponent* could be seen clearly. Each component of an *ImageSelector* contained a single image that was one of the three choices for a given word to be remembered. Objects to be remembered that were to be placed around the palace were known as *Placeables* (see Fig. 5). After the image selection process was complete for an item to be remembered, a chosen image was placed on a blank black background of a flat object with the word associated with it and this new *Placeable* object floated in front of the participant until interacted with. The flat slate object was held physically by the user and was able to be manipulated by the participant using the held VR controllers. *Placeables* were able to be picked up and manipulated naturally. Pressing and releasing the grip trigger button on the controllers was a natural motion just like picking up or dropping a real object, and so it lent more immersion to the simulation. If at any point a *Placeable* was dropped, thrown, or placed in some accidental position, as long as the placement had not been confirmed, participants could use a certain button press on their controllers to bring the *Placeable* back to be floating in front of them once more, like when it spawned.

Once the *Placeable* was placed where the participant wished it to be, a button on the controller was used to confirm the placement. The *Placeable* then played a chiming sound in a 3D fashion coming from the direction where it had been placed in the virtual environment relative to the participant’s position. Once placement was confirmed, the *Placeable* could not be moved, and a new image selection process for the next word in the word list to memorize began as managed by the *ExperimentManager* and a newly spawned *ImageSelector*. Upon completion of the image selection process, a new *Placeable* spawned like the previous, in front of the user to be grabbed and placed. The process repeated until the list of items to be remembered was exhausted.

### Pilot study

A pilot study was conducted to test all aspects of the virtual memory palace system to prepare and refine the system accordingly for the main experiment. The main reasons for conducting the pilot study were to:

- Determine the appropriate amount of time needed to complete each section of the experiment,
- Determine if the virtual memory palace is too large or small for participants to realistically traverse and complete the tasks,
- Determine if the architectural layout of the palace should be altered in some way (e.g., new pathways where there are none, or to remove pathways through the memory palace that existed before, etc.),
- Verify that the size of the word lists in the pre-test and post-test phases were appropriate for the tasks given to participants, and
- Ensure that the process of getting a new word, selecting an image for it, and placing its representative object in the memory palace, was intuitive, and straightforward to allow participants to complete their tasks quickly and efficiently (i.e., without them getting distracted from their goal of memorizing the items).

### Main experiment

The main experiment consisted of four phases: pre-test; participant training phase; VR MoL phase; and post-test, as described in the following sections.

#### Pre-test

Using other similar studies as a framework, participants first completed a spatial cognitive ability test consisting of five training mental rotation tasks and ten testing mental rotation tasks (Shephard and Metzler 2018). The percentage of correct answers and the average time taken per mental rotation task are recorded. This testing was done to collect information that might help explain differences in participant performance during use of the MoL technique. In another study, it was suggested that those who are better at completing mental rotation tasks also tend to have better recall (Vindenes et al. 2018), so the spatial cognitive ability of participants was deemed important.

After this test, 30 randomly selected words of high imageability were presented to each participant from a word pool provided in Madan et al. (2010). Participants were given 15 minutes to memorize as many words as possible, in order if possible, using any memory strategy they wished. Either when the time was up or they felt ready, participants were then moved to a separate testing area where they were asked to write down as many words as they could recall, as closely to the original ordering as possible. Participants were moved to a separate area to avoid testing bias, as it has been shown that information can be more easily recalled in the location in which it was learned (Godden and Baddeley 1975). The written recall test had a maximum time limit of 5 minutes, and participants were not given any feedback on their performance that might influence other parts of the experiment. Words spelled incorrectly were marked as correct.

#### Participant training phase in the VR environment

“As the MoL is known to be a complex mnemonic technique (Yates 1999), it was important to allow participants to become familiar with the technique and the memory palace before performing tasks in the environment. To support this learning, each participant was introduced to what the MoL entailed using an explanation of the technique from Huttner and Robra-Bissantz (2017), paraphrased and used by Legge et al. (2012), and originally taken from Yates (1999). This explanation is as follows from Huttner and Robra-Bissantz 2017; Yates 1999.

‘In this method, memory is established from places and images. If we wish to remember an object, we must first imagine that object as an image, and then place it in a location. If we wish to remember a list of objects, then we must make a path out the many locations. The easiest way would be to imagine a familiar environment and place the imagined objects inside it. Then, you can pick up the objects as you imagine navigating the environment, thereby remembering the object list in order.”

After being told about the technique, participants were encouraged to ask questions. Through encouraging participants to use the MoL technique with direct instruction and thorough explanation, it was hoped to increase compliance in participants, which has been shown to be a problem in MoL experiments with particularly older people (Verhaeghen and Marcoen 1996). When participants felt confident that they understood the MoL technique, they were introduced to the virtual reality equipment prepared to simulate a virtual memory palace.

Participants were given 10 minutes to learn how to use the virtual reality system, so that they were aware of how to grab and place objects, as well as how to walk around in the virtual environment and use the controllers. Time was allotted for participants to explore each of the rooms and areas in the memory palace. Participants were allowed to take more than 10 minutes for this part of the experiment if they felt that they needed it. Familiarity with the memory palace to be used in the MoL has been shown to be a significant factor for better performance in memory recall in several studies (Reggente et al. 2019; Caplan et al. 2019; Jund et al. 2016). It is important to note that participants in the experiment conducted were given very little time to train and prepare for using this technique in comparison with other studies where multiple weeks or a few days were provided for getting familiar with the MoL (Huttner et al. 2019; Bhandari 2019; Liu et al. 2019; Huttner et al. 2018; Huttner and Robra-Bissantz 2017).

**Fig. 9:**
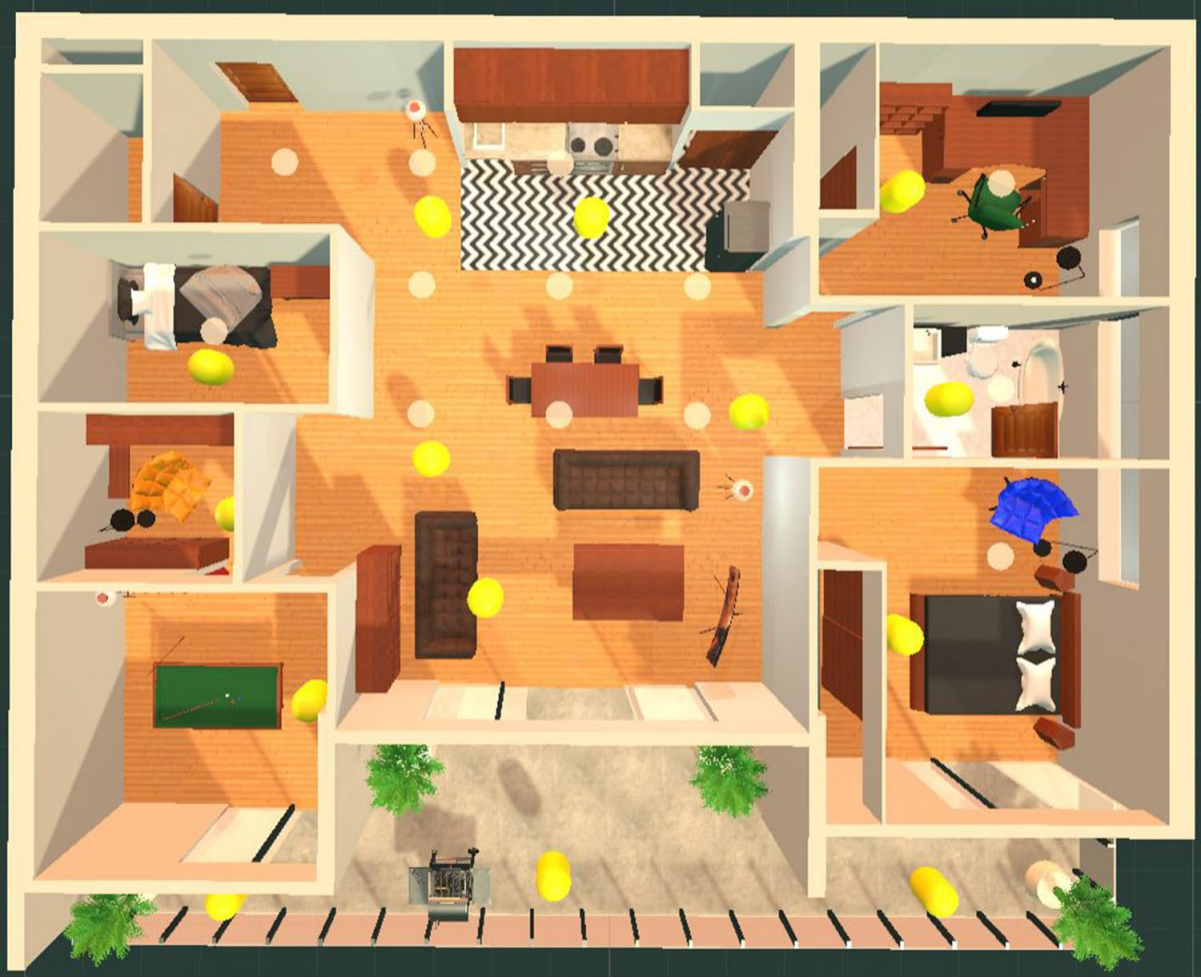
Positions of *Collectibles* throughout the memory palace

As purposeful navigation has been suggested to be more memorable than simply wandering the memory palace without a specific aim (Reggente et al. 2019; Bhandari 2019), 15 floating collectible items were placed around the palace for participants to find and collect by touching them (see Fig. 9). With the items being scattered throughout the palace, collecting them allowed participants to explore every room thoroughly. Collectibles have been successfully used for exploration of memory palaces in other studies (e.g., Reggente et al. 2019; Bhandari 2019), and they follow the principle of trailblazing (Darken and Sibert 1993; Bhandari 2019) by creating a specific path through the memory palace in which a given participant does not need to explore any given area twice.

The phase ended when either time ran out or the participant felt comfortable with the memory palace and the controls of the virtual reality environment.

When exploration of the environment was complete, the participant was then placed in a very short version of the main VR MoL Phase of the experiment. This shortened version involved only 5 words *(Placeables)* (none of which were on the lists of words to actually memorize). This portion of the training phase was intended to introduce participants to the process of selecting images and manipulating Placeable items that represented the words to be memorized. The purpose of this training was to ensure that each participant met the required competency level in the VR environment and no additional learning was required for them to perform the main experiment.

#### VR MoL phase

Similar to the way the pre-test memorization phase was conducted, participants were given 15 minutes to memorize another set of 30 words randomly chosen from the same pool of high imageability words, without choosing any of the words used in the pre-test memorization phase. However, unlike in the pre-test memorization phase, participants made use of the MoL in the virtual environment they had explored in order to memorize the new list of words they were given.

Words were given to them one at a time to present them in a specific order like in the pre-test, to encourage memorizing how they were placed in the MoL as a path through the structure from the first word placed to the last word placed. Keeping track of a path when using the MoL to place and recall memories is integral to the traditional technique, allowing people to theoretically memorize items in their original order with more accuracy than with other memorization strategies (Yates 1999).

When a word was presented to a participant to memorize, the text was shown in front of them with three different images above it that matched the word (see Figure 8). Participants were able to use one hand to select an image they felt they would like to use to memorize a word by simply moving their hand into the image. The other two images would disappear, and the chosen image would be attached to a newly spawned object that looked like a floating black slate. Under the image, the new object also had the word in question to memorize, written on it. The participant could then reach out and grab the black slate object (*Placeable*), and place it in a memorable location in the memory palace.

The motivation behind allowing participants to choose an image that they feel matches with each given word is to allow personalization of items to be remembered. Studies have shown that personalization of how memorized items are displayed can lead to improved recall (Bhandari 2019; Peeters and Segundo-Ortin 2019). This is something often done by memory champions but rarely or never done before in virtual MoL research studies.

When a participant placed an item, it played a 3D sound from the location it was placed in, sounding somewhat like the ring of a bell. Sounds were played to increase sensory involvement in the task of placement. It has been shown that providing more audio, body movement requirements, tactile feedback, and other sensory information to the user can increase immersion and presence in a simulated environment (Sanchez-Vives and Slater 2005; Kong et al. 2017), and as shown in other studies, further immersion leads to better memory recall (Huttner et al. 2019; Krokos et al. 2019; Huttner and Robra-Bissantz 2017).

All throughout the virtual simulation, participants saw a simulated representation of their hands when they looked at themselves, because certain research has shown this will further increase immersion in the environment (Sanchez-Vives and Slater 2005) and thus, increase memory recall accuracy (Huttner et al. 2019; Krokos et al. 2019; Huttner and Robra-Bissantz 2017).

Participants were asked to leave the simulated environment when all items had been placed and the participants felt comfortable with remembering what had been placed, or the time to complete the memorization process had expired.

#### Post-test

Finally, the participants were moved to the same testing area that they were tested in before, and there they were asked to try to recall all 30 of the words that they tried to memorize in the memory palace, in order if possible. Like the pre-test, the post-test took a maximum of 5 minutes, and participants did not know their results afterward. When finished with the post-test, participants were given a closing questionnaire (see Appendix A). These questions were chosen due to their similarity to those posed to participants in other similar studies (Legge et al. 2012; Huttner and Robra-Bissantz 2017; Caplan et al. 2019; Reggente et al. 2019).

### Internal validity

To ensure that the experiment was measuring what it was intended to effectively, various measures were taken:

- Experimental data were recorded electronically by automated scripts. This was done to minimize human error when recording information.
- Participants were given as much time as they required to get comfortable with the VR environment, movement using the motion sensors, and making use of the controls in the simulated memory palace. This was done to bring all participants up to the same level of comfort, competency and to reduce any bias toward participants with more VR experience. Participants started the VR MoL phase of the experiment only when they were fully comfortable and ready to do so.
- The short training period participants experienced to place five items in the memory palace before commencing the VR MoL phase of the experiment aimed to reduce bias toward those that learned the VR environment’s interface faster than others.
- A pilot study was conducted to ensure that the main experiment was designed properly to address the research question.

We believe that bias was reduced through these activities to ensure clear alignment between the experiment and the results achieved.

### External validity

The results of this work can be generalized to multiple domains in which a VR-based MoL technique could be useful to memorize non-spatial information for a given purpose. Other studies such as Huttner et al. (2018), Huttner et al. (2019), Huttner et al. (2015), have discussed the potential of an effective VR-based MoL technique before. Some example potential uses of this system could be:

- In universities, and other educational institutions, where students need to memorize large amounts of information (e.g., medical school). Professors could also use this system to help them memorize lesson material;
- to help those who have lost some of their memorization abilities, either through aging or other reasons. Such a system could be used to maintain and/or improve their memorization abilities, especially in a long-term capacity. The MoL has been suggested to be quite effective for long-term memory; (Huttner and Robra-Bissantz 2017; Optale et al. 2010).
- to help people remember particularly difficult to recall memories, as suggested in Dalgleish et al. (2013); and
- to provide a powerful, easy to learn memorization technique that removes the original complexities of the MoL such as extensive training. Our VR MoL system could be useful to just about anyone who does not already use highly effective memorization techniques in their daily lives.

### Analysis

As done with multiple other similar studies (Mccabe 2015; Huttner et al. 2015, 2019; Liu et al. 2019; Huttner et al. 2018; Huttner and Robra-Bissantz 2017), originally proposed and used by the study (Legge et al. 2012), memory recall performance for the pre-tests and post-tests were calculated by determining *lenient* and *strict scores* of participants. The lenient score is a percentage of simply how many words the participant recalled correctly divided by the total number of words to remember. On the other hand, the strict score is calculated using the *levenshtein distance*, an algorithm for determining how many changes must be made to a list of recalled words to make the list identical to the original list (Huttner et al. 2019). This algorithm calculates the minimum costs of transforming one sequence (e.g., a string or an array of terms) into an original one (Huttner et al. 2019). The algorithm includes three basic operations: *replace, delete* and *insert*. Every time the algorithm has to use one of them, a counter increments the costs of transformation by one. In the end, the minimum costs are returned. For instance, the original sequence is *table, spoon, fork, apple, banana,* while the user’s input was *spoon, fork, apple, banana, table*. In this case, the order is almost perfect except for the term *table*. The *levenshtein distance* then deletes *table* and adds it at the beginning of the sequence. Hence, two operations were performed (deletion and insertion) and the cost of transforming the sequence is two. The strict score is a measure of whether words were remembered in the correct order and is computed using the following formula (Eq. 1):

1 $$\begin{aligned} strict\_score = 1~ - ~\frac{lev(u,o)}{max} \end{aligned}$$

where *lev*(*u*, *o*) returns the *levenshtein* costs of the user input sequence *u* and the original sequence *o*. The value *max* represents the maximum amount of operations that might be necessary to transform any given sequence of terms into the original one Huttner et al. (2019).

Standard descriptive statistics including *t* tests, boxplots, and a two-way ANOVA were also computed to determine the performance of participants.

## Findings (analysis and evaluation)

### Mental rotation task results

The first part of the experiment participants completed was the mental rotation tasks to measure their spatial cognitive ability. These results are shown in Table 2. The mean performance across all 11 participants was 82.73% with a *SD* = 8.06. The average time taken per mental rotation task was 4.90 seconds with a *SD* = 2.39.

No significant patterns were observed to correlate the mental rotation task results with how well a given participant did with the VR MoL phase of the experiment. In contrast, higher performance on mental rotation tasks, and therefore better spatial cognitive abilities, were observed to generally lead to better recall in participants in Vindenes et al. (2018). It is hypothesized that if this experiment had been conducted with more participants, a pattern may have emerged to follow this trend.

**Table 2:** Participant mental rotation task performance and timings

Performance (correct mental rotation task answers (%))				Time taken per mental rotation task (seconds)			
Mean	Min	Max	Standard deviation (σ)	Mean	Min	Max	Standard deviation (σ)
82.73	70	93	8.06	4.90	2.23	9.35	2.39

### Traditional studying (pre-test) phase results

**Fig. 10:**
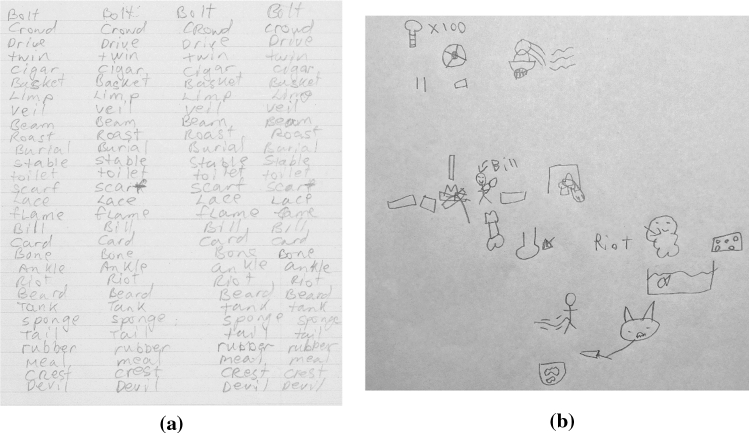
**a** Participant P6’s repetition strategy to memorize the word list; and **b** Participant P9’s drawing of small images of each object which provided the framework for a story to be created that linked all the images together

After completion of their mental rotation tasks, participants were given 15 minutes to memorize a list of 30 words, using a single piece of scrap paper if they wanted to draw or write anything down to help with whatever mnemonic technique they decided to use. Participants almost always used all the 15 minutes allotted to them for studying the list. Various studying methods were used for this phase, as indicated by the participants through the scrap paper given to them and the questionnaire at the end of the experiment where people reported what strategies they used. Participants P4, P6, P8, P10, and P11 used repetition, trying to remember by repeating the words to themselves out loud and by writing them down over and over. P1, P7, and P9 created a story out of the words instead. Figure 10a, b illustrate these strategies, respectively. P2, P3, and P5 used hybrid approaches by making associations between words and images, linking two words into a single combined image, or creating an acronym out of different words to associate.

### VR MoL studying (post-test) phase results

**Fig. 11:**
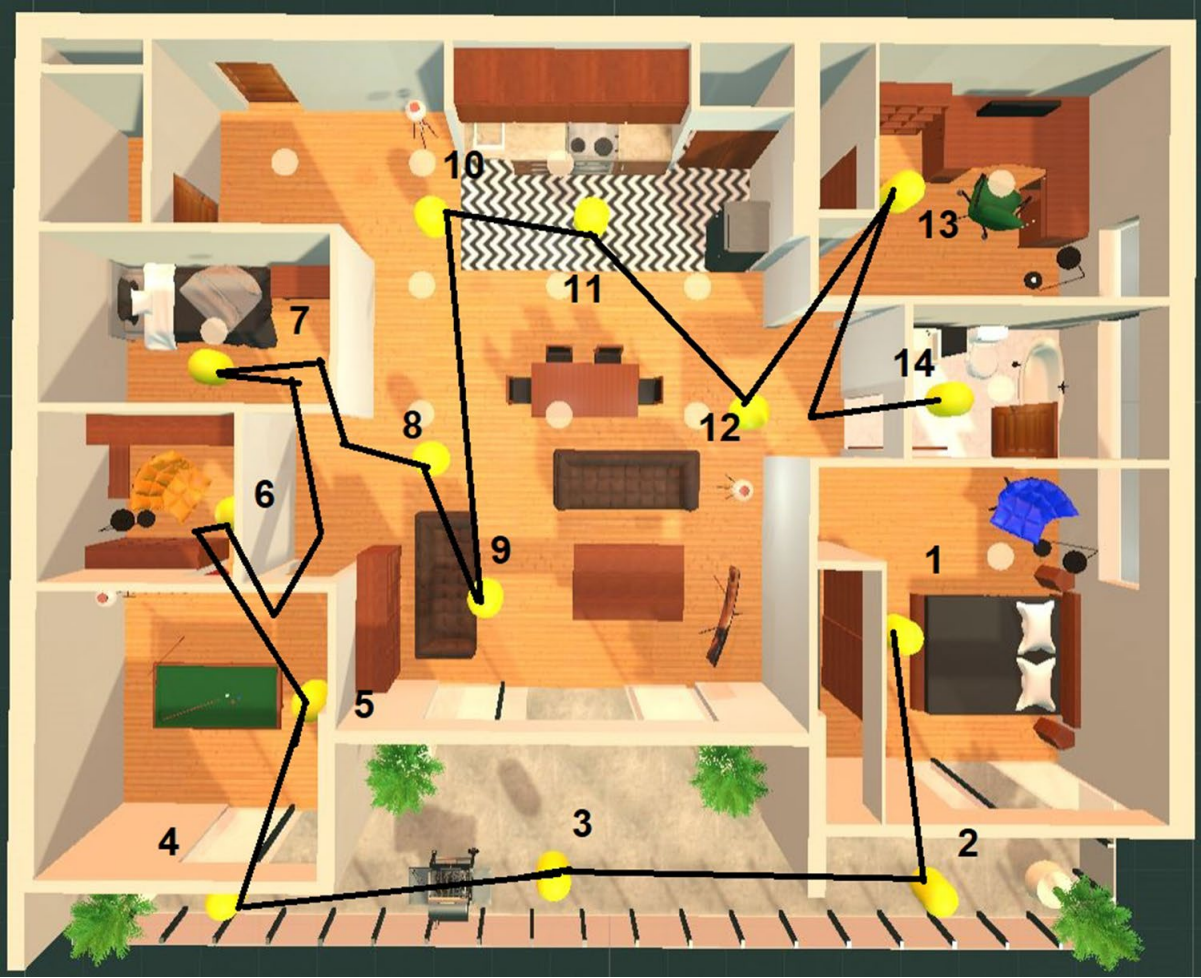
Path taken by Participant P4 during the Collectibles phase

When participants collected each of the Collectible objects in the memory palace, the order that they collected each of these items was recorded. Figure 11 illustrates an example path that a participant (P4) took. On average, it took approximately 3 minutes and 3 seconds for participants to collect all of the Collectibles in the memory palace during the training phase of the experiment. In the first week, this average time was 3 minutes and 26 seconds, and in the second week, it was 2 minutes and 34 seconds. Participants in the second week of the experiment reported feeling much more comfortable with the system than in the previous week and predictably took less time to complete the exploration of the memory palace through collecting Collectibles. Summary statistics are provided in Tables 3 and 4.

**Table 3:** Summary statistics for time taken for participants to collect all Collectibles and Placeables in Week 1

Time taken to collect all Collectibles (Week 1) (seconds)				Time taken to collect all Placeables (Week 1) (seconds)			
Mean	Min	Max	Standard deviation (σ)	Mean	Min	Max	Standard deviation (σ)
206.12	117.10	360.40	103.72	637.47	567	729.7	61.79

**Table 4:** Summary statistics for time taken for participants to collect all Collectibles and Placeables in Week 2

Time taken to collect all Collectibles (Week 2) (seconds)				Time taken to collect all Placeables (Week 2) (seconds)			
Mean	Min	Max	Standard deviation (σ)	Mean	Min	Max	Standard deviation (σ)
154.30	95.20	283.50	87.73	429.22	429.22	516.90	106.33

On average, it took 10 minutes and 55 seconds for participants to place all of the Placeable items in the VR MoL phase. In the second week, it took an average of 8 minutes and 10 seconds. The decrease in time is likely due to the participants getting more comfortable with the VR MoL system, as they stated was the case after the experiments in the second week. Participants felt comfortable with the remaining time given to them for reviewing their placements (the remainder of their 15 minute time limit to complete the VR MoL phase), and no participant felt the need to rush the procedure. Some participants did not need all of the allotted time and left the environment early if they wished to.

### Overall mental recall results

Tables 5 and 6 present the lenient and strict score results for week 1 and week 2, respectively. These scores were calculated based on the lists of words written down by participants trying to recall the word lists they were asked to memorize in the pre-test and post-test phases.

**Table 5:** Week 1 Lenient and Strict Scores

Week 1 Lenient Recall Scores					Week 1 Strict Recall Scores				
	Mean	Min	Max	Standard deviation (σ)		Mean	Min	Max	Standard deviation (σ)
Pre-test	62.55	27.00	100.00	24.01	pre-test	39.36	3.00	90.00	32.92
Post-test	82.91	53.00	100.00	15.99	post-test	23.18	3.00	77.00	23.89

**Table 6:** Week 2 Lenient and Strict Scores

Week 2 Lenient Recall Scores					Week 2 Strict Recall Scores				
	Mean	Min	Max	Standard deviation (σ)		Mean	Min	Max	Standard deviation (σ)
Pre-test	63.44	30.00	100.00	26.64	pre-test	42.56	10.00	100.00	30.10
Post-test	85.67	47.00	97.00	16.10	post-test	20.44	7.00	60.00	22.51

**Fig. 12:**
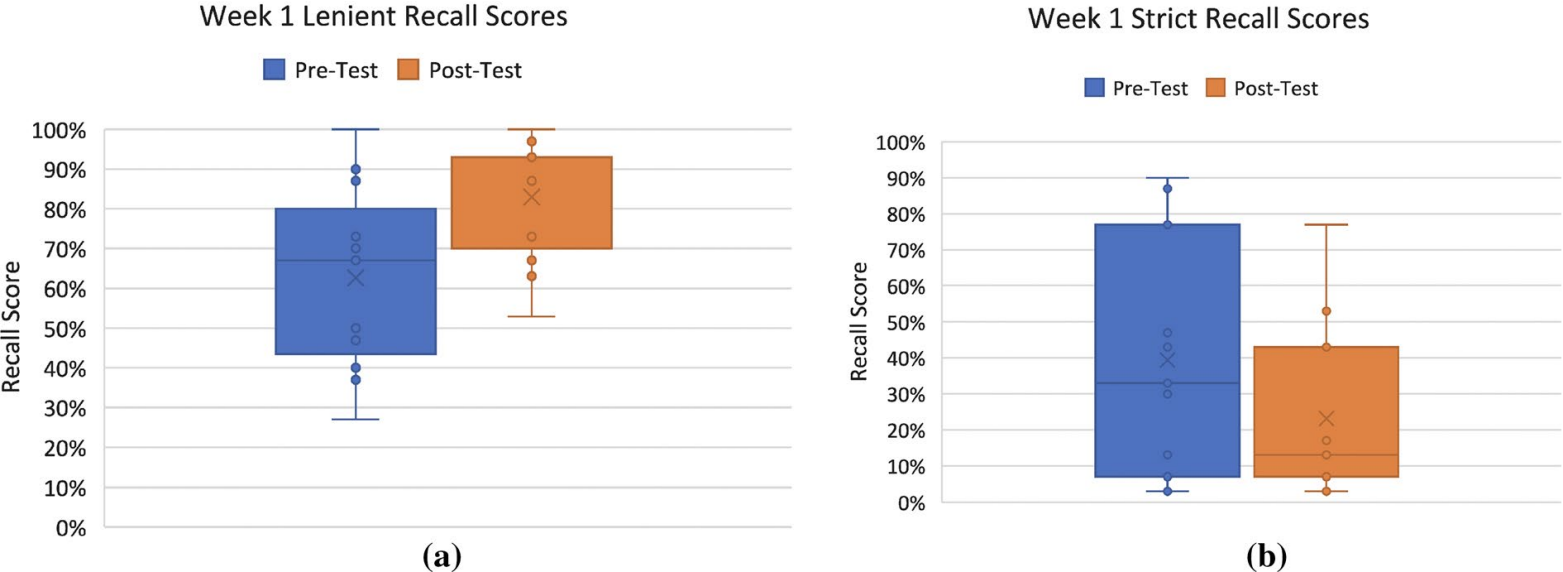
**a** Week 1 Lenient Recall Scores and **b** Week 1 Strict Recall Scores

**Fig. 13:**
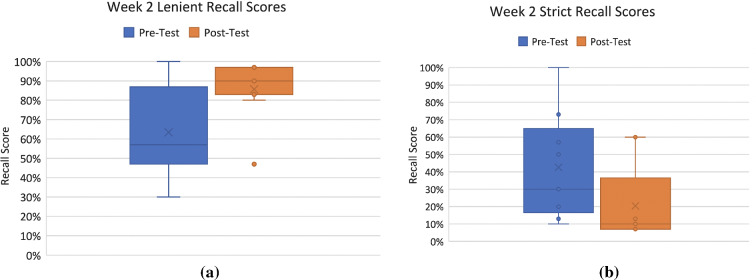
**a** Week 2 Lenient Recall Scores; and **b** Week 2 Strict Recall Scores

Figure 12a, b shows the distribution of test scores in both pre-test and post-test for lenient and strict scoring, for the first week of the experiment, displaying a visible increase in lenient scores from pre-test to post-test conditions, while displaying a decline in strict score from pre-test to post-test. Similar trends, albeit more pronounced, are presented in the boxplots in Figure 13a, b for week 2.

Several *t* tests were conducted for lenient and strict recall scores for week 1 and 2 based on the pre-tests and post-tests. The results from week 1 lenient recall scores on the pre-test (*M* = 62.55, *SD* = 24.01) and post-test (*M* = 82.91, *SD* = 15.99) memory task indicate that the increase in number of words remembered was statistically significant, *t*(20) = − 2.34, *p* = 0.014. The post hoc statistical power for this *t* test was calculated which revealed 41.2% observed power (given the observed effect size [Cohen’s *d*: 0.98], probability level of 0.05, and a sample size of 11). The formulas presented in Eqs. 2, 3, 4, 5, 6, and 7 were used in the calculation for the post hoc power for the *t* tests (Cohen 1988).

#### Beta function

2 $$\begin{aligned} B(x,y) = \int _{0}^{1} t^{x-1}(1-t)^{y-1}dt \end{aligned}$$

#### Cohen’s d effect size for a ***t***-test

3 $$\begin{aligned} d = \frac{|\bar{x}_1 - \bar{x}_2|}{\sqrt{(\sigma ^2_1 + \sigma ^2_2)/2} } , \end{aligned}$$

where $\bar{x}_1$ and $\bar{x}_2$ are the means of group 1 and group 2, and $\sigma _1^2$ and $\sigma _2^2$ are the variances of group 1 and group 2.

#### Gamma function

4 $$\begin{aligned} \varGamma {(z)} = \int _{0}^{\infty }t^{z-1}e^{-t}dt \end{aligned}$$

#### Lower incomplete beta function

5 $$\begin{aligned} B(x;a,b) = \int _{0}^{x}t^{a-1}(1-t)^{b-1}dt \end{aligned}$$

#### Noncentral t-distribution noncentrality parameter

6 $$\begin{aligned} \delta = d \sqrt{\frac{n_1n_2}{n_1+n_2}} , \end{aligned}$$

where *d* is the Cohen’s d effect size, and $n_1$ and $n_2$ are the sample sizes for group 1 and group 2.

#### Regularized lower incomplete beta function

7 $$\begin{aligned} I_{x}(a, b) = \frac{B(x;a,b)}{B(a,b)} , \end{aligned}$$

where the numerator is the lower incomplete beta function, and the denominator is the beta function.

Similarly, the results from week 2 lenient recall scores on the pre-test (*M* = 63.44, *SD* = 26.64) and post-test (*M* = 85.67, *SD* = 16.10) memory task indicate that the increase in number of words remembered was statistically significant, *t*(16) = -2.142, *p* = 0.024. The post hoc statistical power for this *t* test was calculated which revealed 44.7% observed power (given the observed effect size [Cohen’s *d*: 1.05], probability level of 0.05, and a sample size of 11).

However, the results from week 1 strict recall scores on the pre-test (*M* = 39.36, *SD* = 32.92) and post-test (*M* = 23.18, *SD* = 23.89) memory task indicate that there was not a statistically significant improvement or drop in performance. This was also the case for week 2 strict recall scores on the pre-test (*M* = 42.56, *SD* = 30.10) and post-test (*M* = 20.44, *SD* = 22.51) memory task, which indicate that there was no statistically significant improvement. In summary, the *t* test results indicate that in both weeks of the experiment, there was a significant improvement in lenient scores during the post-test.

A two-way ANOVA with repeated measures was also performed to analyze the effect of Trial (week 1 vs. week 2) and Memorization Strategy (Traditional vs. VR MoL) on memory recall (using lenient scoring). The following assumptions were met for the ANOVA: (1) *Independence of variables*: The two variables for testing are independent from each other; (2) *Homoscedasticity*: The variance in this two-way ANOVA was homogenous, that is, the variation around the mean for each set did not vary significantly for the groups; and (3) *Normal distribution of variables*: The participant’s scores followed a normal distribution pattern.

The test for Homoscedasticity was conducted using Barlett’s test for homogeneity of variances (Barlett 1937). The formula is presented in Equation 8.

#### Barlett’s test for homogeneity

8 $$\begin{aligned} B = \frac{(N-k)ln(S^{2}_p)-\sum _{i = 1}^{k}(n_i-1)ln(S^2_i)}{1+\frac{1}{3(k-1)} (\sum _{i = 1}^{k}(\frac{1}{n_i-1})-\frac{1}{N-k})} , \end{aligned}$$

where *n*: the total number of observations across all groups, *k*: the total number of groups, *ln*: the ‘natural log,’ $s_2$: The pooled variance, $n_j$: The number of observations in group j, $s_j^2$: The variance of group jThe results from Barlett’s test were *B*: 4.586 and the *p*-value of: 0.095, thus verifying the assumption that the variances were equal across samples.

**Fig. 14:**
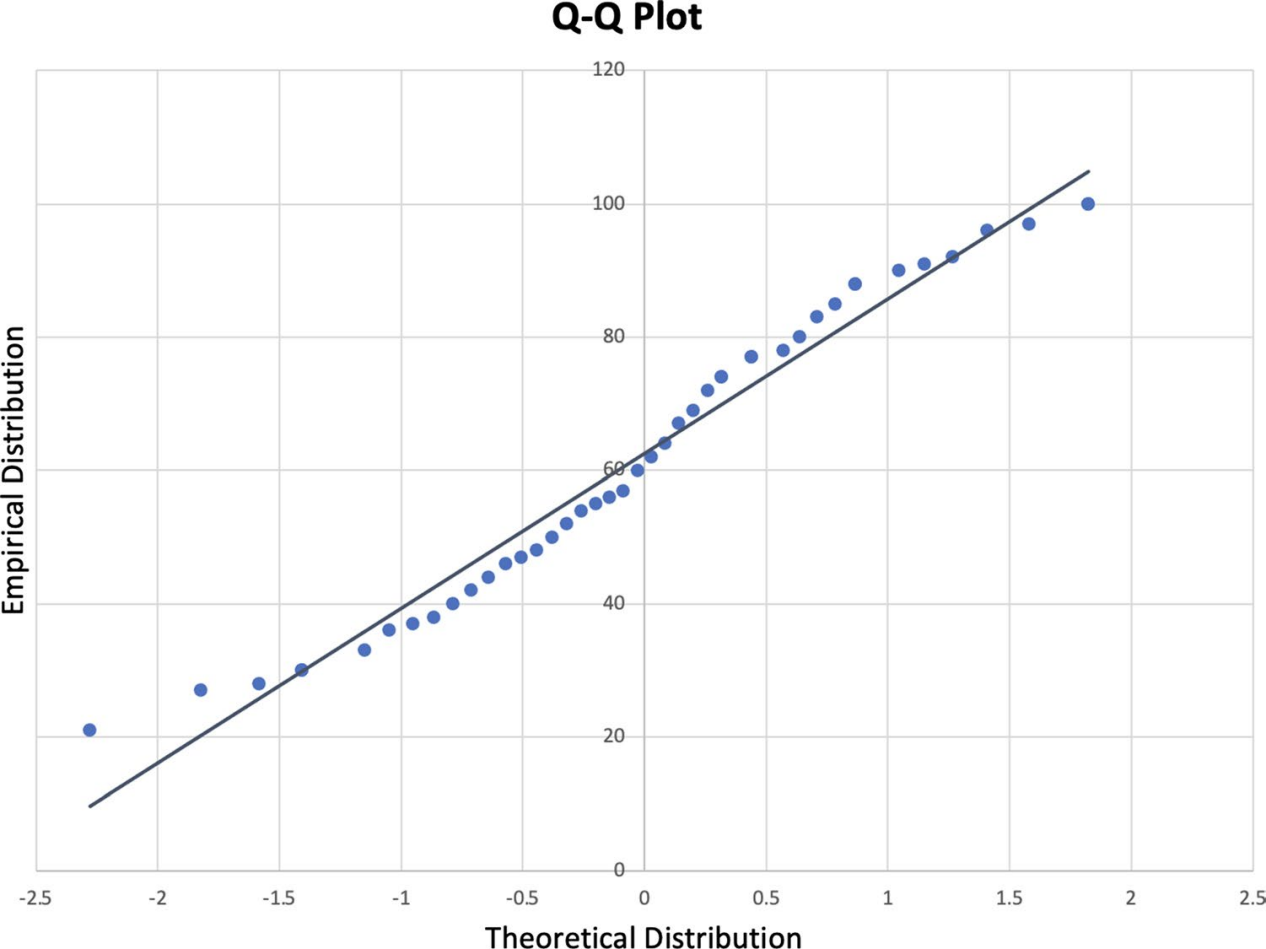
Q-Q Plot

A Q-Q plot and a Shapiro–Wilk test were used to assess the distribution of the participant’s scores. Figure 14 presents the Q-Q plot which visually shows a close approximation to a normal distribution. The Shapiro–Wilk test is commonly used to determine if the data is normally distributed (Shapiro and Wilk 1965). The Shapiro–Wilk formula is shown in Equation 9. The results were: W(472) = 0.952, and *p*-value = 0.258 (i.e., p > $\alpha$ (0.05)), indicating the participant’s scores followed a normal distribution.

#### Shapiro–Wilk normality test

9 $$\begin{aligned} W = \frac{(\sum _{i = 1}^{n}a_{i}x_{(i)})^2}{\sum _{i = 1}^{n}(x_i-\bar{x})^2 } , \end{aligned}$$

where

- $x_{(i)}$ is the *i*th order statistic;
- $\bar{x} = (x_1 + \cdots + x_n)/n$ is the sample mean,
- the coefficients $a_i$ are: $(a_1, \ldots , a_n) = \frac{m^TV^{-1}}{C}$, where
- $$C = ||V^{-1}m|| = \sqrt{ (m^TV^{-1}V^{-1}m)}$$
- and $$m = (m_1, \ldots , m_n)^T$$

The results of the ANOVA showed a significant main effect for memorization strategy, *F*(1, 40) = 9.914, *p* = .003 indicating the VR MoL strategy performed well-above traditional memorization techniques. Table 7 presents these results. The *p*-value for the interaction between trials (i.e., week 1 and 2) and memorization strategy (traditional and VR MoL) was 0.914. This was not statistically significant at the $\alpha = 0.05$ level. The *p*-value for trial was 0.450, which was also not statistically significant at the $\alpha = 0.05$ level. The *p*-value for memorization strategy was 0.003 which was statistically significant at the $\alpha = 0.05$ level. A post hoc statistical power calculation was performed on the ANOVA for main effect for memorization strategy which revealed a 94% power for this study as shown in Equations 10–14. Using the root-mean-square standardized effect (RMSSE) (Steiger 2004), as presented in Equation 15, the effect size was 0.727.

**Table 7:** Two-factor ANOVA with repeated measures by trial and memorization strategy on memory recall

Source of Variation	SS	d*f*	MS	*F*	*P*-value	*F * crit
Trial: week 1 or 2	250.568	1	250.568	0.583	0.450	4.085
Memorization strategy: Traditional or VR MoL	4261.114	1	4261.114	9.914	0.003	4.085
Interaction	5.114	1	5.114	0.012	0.914	4.085
Within	17191.636	40	429.791			
Total	21708.432	43

10 $$\begin{aligned} Power& = \phi \left\{ {-Z_{1-a/2}+\frac{\varDelta }{\sqrt{\sigma ^{2}_{1}/n_1+\sigma ^{2}_{2}/n_2}}} \right\} \end{aligned}$$

11 $$\begin{aligned}& = \phi \left\{ {-(1.96)}+\frac{3}{\sqrt{2^2/11+2^2/11}} \right\} \end{aligned}$$

12 $$\begin{aligned}& = \phi \left\{ {1.558} \right\} \end{aligned}$$

13 $$\begin{aligned}& = 0.94 \end{aligned}$$

14 $$\begin{aligned}& = 94\% \textit{power} \end{aligned}$$

#### RMSSE for measuring the ANOVA effect size

15 $$\begin{aligned} d = \sqrt{\frac{\sum _{j = 1}^{k}d^{2}_j}{k-1}} , \end{aligned}$$

where

- $$d_j = \frac{u_j-u}{\sigma } \approx \frac{ {\bar{x}_j}-\bar{x} }{\sqrt{MS_E}}$$

These results indicate that memorization strategy was the only factor that had a statistically significant effect on memory recall, and there were no interaction effects, that is, exposure to the trial in week 1 did not have a statistically significant impact on the participant’s performance in week 2.

### Qualitative findings based on the questionnaire

This section presents the findings from the questionnaire each participant completed at the end of the experiment. Participants were asked to talk about their pre-test memorization technique, their experience level in VR, whether they actually used the Method of Loci, their confidence in their answers in the pre-test versus the post-test, how immersive they found the environment, whether they knew about the MoL beforehand, and whether they would use the system in a real-life example to study something. The questionnaire was given out during the first week of the experiment only. In the second week, participants were asked if they felt more comfortable using the system, to which all participants agreed that they did. A summary of the questionnaire’s results is presented in Table 8.

**Table 8:** Summary of questionnaire results

Description	%
Believed they used the MoL as instructed	100
Would use the system in a real-life scenario	91
Very immersed	82
Very little/No experience with VR	73
Very confident in post-test	72
Very confident in pre-test	46
Used repetition to remember words in pre-test	45
Had a little prior knowledge of the MoL	36
Somewhat experienced with VR	27
Somewhat confident in pre-test	27
Not very confident in pre-test	27
Created a story to remember words in pre-test	27
Used association to remember words in pre-test	27
Not very confident in post-test	18
Somewhat immersed	18
Somewhat confident in post-test	9

## Discussion

### Traditional studying phase (pre-test)

Between weeks 1 and 2, some participants chose to change the strategy they used to memorize the words. The change in strategy often led to much improved results compared to the week prior. For example, participant P3 used an acronym association technique in the first week, and achieved an accuracy of 67% (lenient scoring) and in the second week, they used an association between drawn images and the words that they represent, resulting in an accuracy of 93% (lenient scoring). Thus, P3’s performance improved by 26%.

Other participant pre-test results also improved significantly by changing their strategies. Several mentioned after the experiment in the second week that they were inspired by using the MoL in the first week and wanted to change their traditional studying technique to include more connections with pictures rather than just text like with repetition and acronym studying strategies.

### Training in the VR MoL environment

In the first week of the experiment, participants needed some time to get comfortable with the motion sensors from KAT VR to learn how to walk in the simulation. In the second week of the experiment, participants were more eager to move through the environment at faster speeds. Almost all participants seemed to experience increased immersion, sometimes to the point of forgetting that they needed to walk in place rather than normally, leading to nearly bumping into walls of the testing room.

During the Collectibles phase, participants seemed to move in mostly random paths, and no significant patterns were observed.

### Testing in the VR MoL environment

The size of our word list was suitable for our study; however, in a future study, it is recommended to follow the suggested minimum word list size of 40 (or greater) from (Ross and Lawrence 1968). We selected 30 because we were concerned about nausea from the virtual reality environment if participants were in it for too long. Fortunately, in our study, much of this nausea seemed to be negated by the natural walking movements participants could use, as afforded by the KAT VR motion sensors.

It was discovered that there were specific rooms in the memory palace that seemed to be either completely forgotten by participants or very seldom used. The least used parts of the memory palace were the storage room, the smaller of the two bedrooms, and the recreation room (refer to Figure 3). Perhaps one reason why this happened is because the rooms were not as visibly accessible from the large central space in the apartment compared to the office, bathroom, and large bedroom. It seems that an optimal memory palace design would have entrances to each available room always visible from the central space of the environment. This way people would be more encouraged to make use of all rooms available to them.

The image selector used in the experiment by participants to choose which image would be used with created Placeable objects was a concept suggested by another study Peeters and Segundo-Ortin (2019) but entirely new to actual implementation. It was well-received by participants, who enjoyed the degree of personalization in their Placeable items and likely created stronger memory associations with the pictures chosen and their paired words to memorize.

Participant P4 also commented that in both weeks of the experiment, the associations between the image and the word given, in the virtual reality environment, helped a great deal in trying to memorize items. It was common among participants to say that in the recall test they remembered the images of the things they placed long before they remembered what word they were associated with. It is hypothesized that in an optimal scenario where participants could use any image of their choosing for a given Placeable they are studying, that the effect of personalizing the images on improving memory recall would become heightened further.

### VR MoL studying phase (post-test)

When using the Method of Loci in VR, on average, participants had their lenient scores increase by over 20% in the first week of the experiment, as observed in the difference between the means of their pre-test and post-test lenient scores. This means that people usually remembered 20.4% more of the items from the list, and therefore their memory recall was improved significantly compared to when they used traditional studying techniques to memorize words. In the second week, this lenient score improvement rose to 22.2%. The *t* test computed on participant lenient scores indicated that the improvements were statistically significant at the $\alpha$ = 0.05 level.

It is important to note that participants were given very little time (less than half an hour), to familiarize themselves with the MoL and the VR environment to virtually simulate the technique. Nonetheless, it was shown that improved memory recall results can be achieved with very little training and treatment time, at least with the small sample size that was available. Perhaps if participants were given more time to become comfortable with the VR MoL system, then it is postulated that there would be even further increased performance results. Due to limited participants, it cannot be said without doubt that memory recall improvements were accurate and unbiased, but it is believed that the results at least show the feasibility of creating an optimized VR MoL system to help participants learn and use the complicated MoL technique with ease in a short time. The software developed for this study can also be used in the future by other researchers to study with more participants and produce more reliable results that could possibly prove an improvement in memory recall is consistently experienced by participants using the given VR MoL system (or a further optimized one).

There is also a point to be said about the potential improvements to long-term memory as opposed to traditional studying techniques. Perhaps in a future study, participants could be asked if they can remember the words from the pre-test and post-test phases of an attempt they did several weeks ago. It is hypothesized that this would show more favorable results for the MoL, and this idea of long-term memory being improved through the MoL has been suggested by multiple other studies such as Optale et al. (2010). We hypothesize this because in our own researcher observations, we noticed that participants, when recalling words they were tested on from the previous week of the experiment during the second week, believed that they remembered more words from the VR MoL studying phase than the traditional studying phase they had participated in the week before.

Through observing participants try to implement the Method of Loci in the memory palace on their given VR MoL phases of the experiment, it became evident that there were different strategies in which one could use to complete the task despite being told to use a specific mnemonic technique. It is possible that the definition of the Method of Loci is perhaps too loosely defined. While almost all participants experienced improvement in their lenient scores when using the VR MoL environment to study, it seemed from observations that the strict score of participants depended greatly on how they used the Method of Loci, in other words, what was their strategy in using it. It was observed that the range of strategies participants used fell into one of the following categories:

1. Associating items with each other in groups and thinking less about the environmental connections;
2. Associating items to their best locations in the environment while forgetting their order;
3. Placing items in a path through the environment without thinking about specifically where they are placed; and
4. Moving through the environment in a specific path and placing items in their most relevant locations along that path to maintain both environmental associations and the order of the items.

These four categories of strategies are ordered from what seemed to be the least effective to the most effective with regard to achieving the highest lenient and strict scores.

**Fig. 15:**
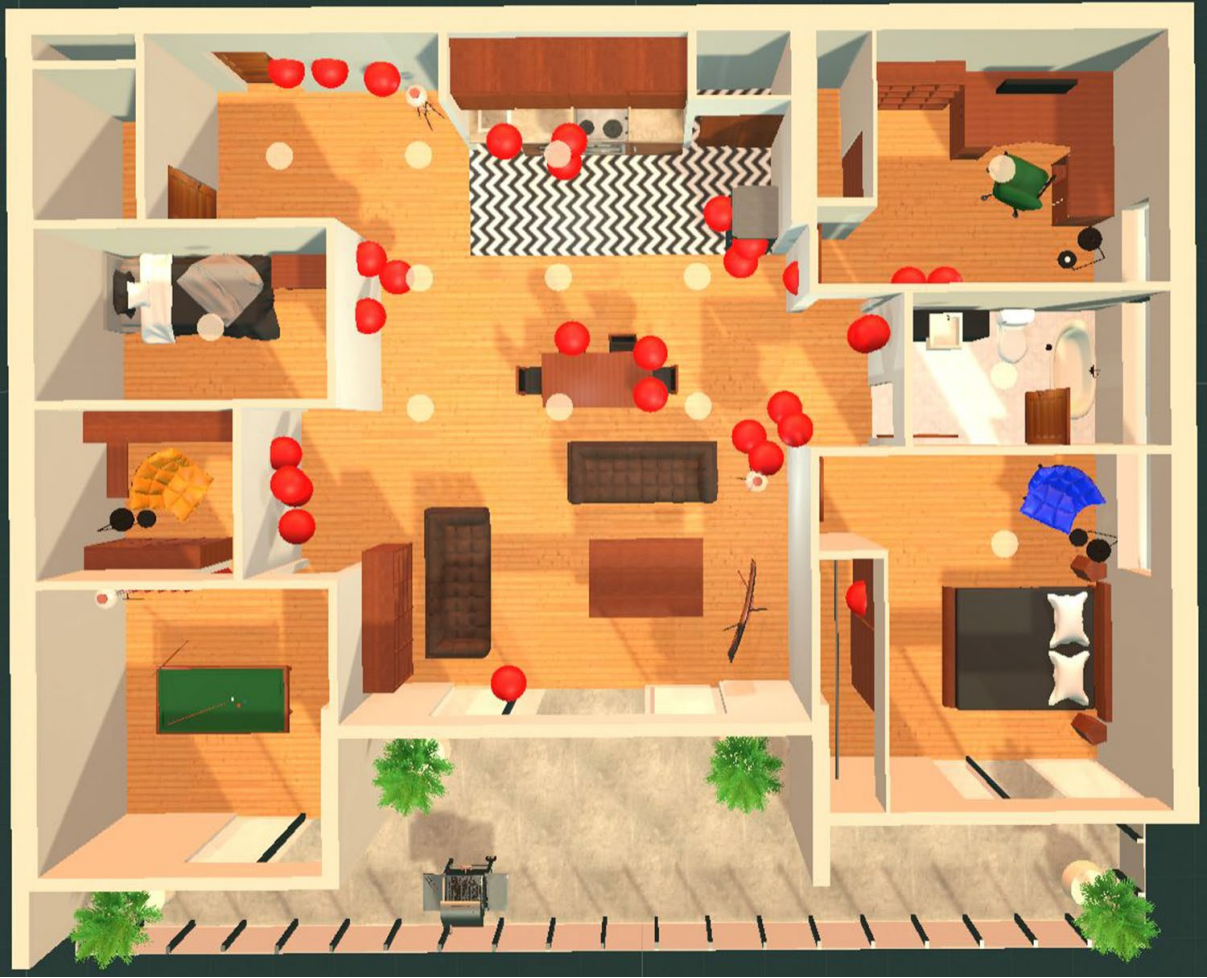
Locations where participant P4 placed their Placeable items in the first week of the experiment (as denoted by the red spheres) (Color figure online)

Participant P4 is a good example when discussing strategies for implementing the Method of Loci. P4 used the first strategy mentioned in the first week, then the last one mentioned in the second week. When they changed their strategy, their strict and lenient score both improved significantly. In the second week, P4 had a lenient score 17% higher than the previous week, and a strict score 47% higher. In the first week, P4 placed almost all of their items in the main room of the apartment with the living room, dining room, and kitchen combined. The placements were not very much inspired by their link to their placement location, as opposed to their link to each other. It was a way of associating the words together, forming them into related groups. Also, being cramped into a single space, the participant did not make use of the full memory palace. There was no visible order to the placements either. The locations where P4 placed items in the first week are shown in Fig. 15.

**Fig. 16:**
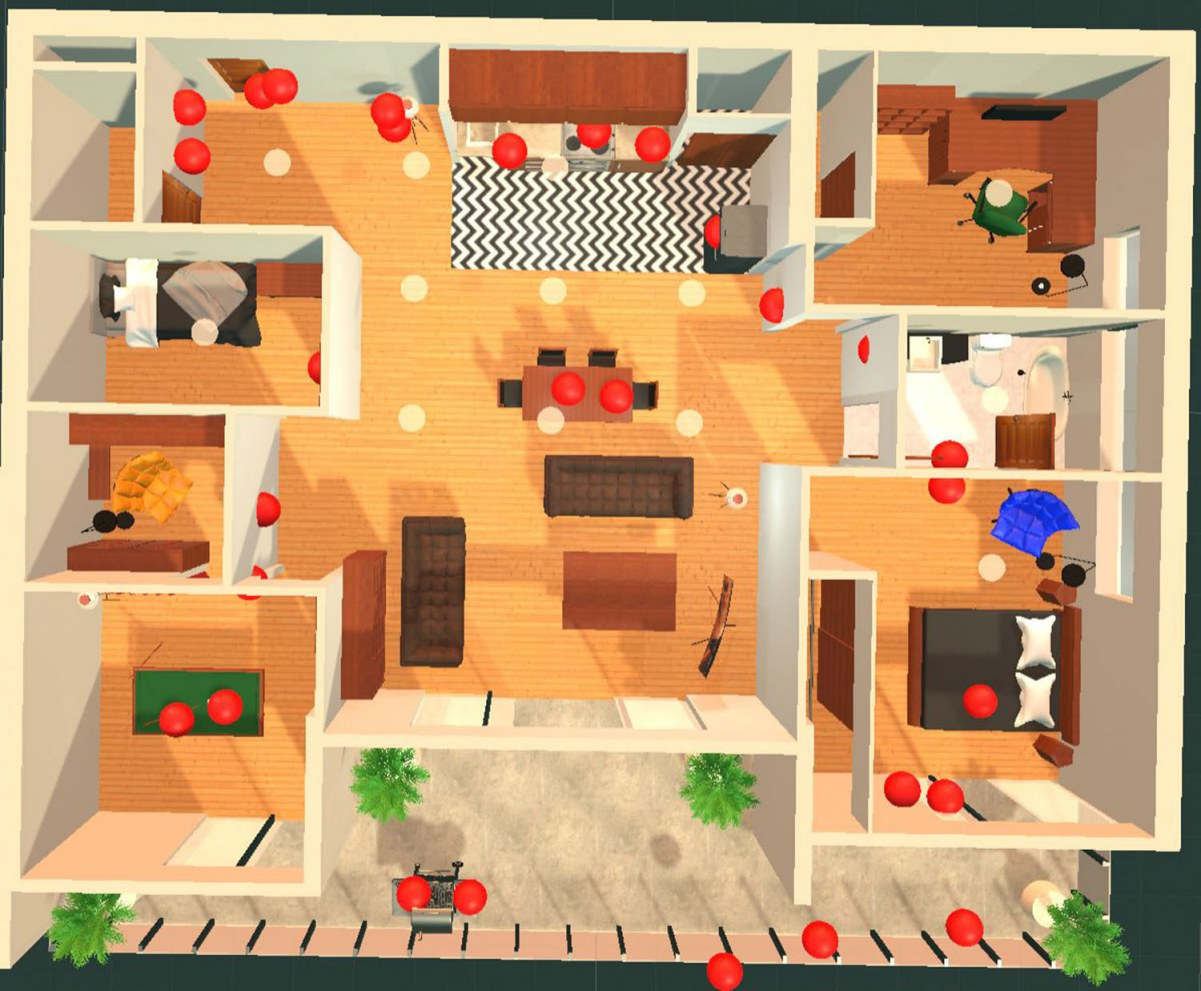
Locations where participant P4 placed their Placeable items in the second week of the experiment (as denoted by the red spheres) (Color figure online)

However, in the second week of the experiment, participant P4 decided to use nearly all of the rooms in the palace, walking in a specific path through the many rooms and placing items as they walked, visibly in the order they were given to them. Rather than placing items according to where they would best fit in the entire palace, instead participant P4 placed them where they seemed to best fit in the current position of their path through the memory palace. For example, when the word ‘pole’ came up, it was placed on a lamp that had a pole in its base next to P4’s current location. Similarly, the word ‘smoke’ was placed off of the balcony’s edge when P4 walked past the balcony at the same time as receiving the word. By doing this, P4 continued to make associations between the environment and the given items, thinking carefully about the current location, while still retaining the order of the items according to a walked path. It may require more thinking and imagination to come up with some kind of connection between a nearby location and the item to be placed (placing the word ‘bullet’ in the bedroom for instance), but that extra thinking could lead to even stronger associations. The locations where P4 placed their Placeables in the second week can be seen in Fig. 16.

Participant results in this study may have become biased due to a lack of counterbalancing from pre-test to post-test to avoid things like learning effects and fatigue. We accepted this possibility but designed the experiment as it is based on the number of participants available to us, which was admittedly quite small due to the pressures of COVID-19 and associated government restrictions. This is also why this study seeks only to prove the feasibility of optimizing the MoL through VR.

After completing our research, we came to realize that a clear comparison between traditional MoL and our new VR MoL environment would have produced more meaningful results. We believe that it is an excellent area for future research, even though in our time constraints, we did not believe we could sufficiently compare the two versions of the MoL fairly, due to the large amount of time that is required to learn the traditional MoL technique. The comparison in how long it takes to learn each version of the MoL could have impactful results especially in encouraging more widespread use of the MoL technique.

### Qualitative findings based on the questionnaire

The qualitative findings were primarily based on the information participants provided in the questionnaire (see Table 8). For example, participants were asked to rate the immersiveness they felt in the environment based on a scale from 1 to 5, where 1 was the least immersive. Two participants rated it as a 3, three as a 4, and six as a 5. For most participants, the presence one felt in the experiment seems to reflect how well they performed in the post-test (as far as their lenient score) after using the VR MoL environment to study the word list given to them. Those who rated the immersiveness lower (1, 2, or 3) tended to have worse performance when compared to those who rated the immersiveness as high (4 or 5). Higher immersiveness leading to better recall is clearly shown to be the trend here, and it is a trend observed in multiple other studies such as Krokos et al. (2019), Huttner and Robra-Bissantz (2017), and Huttner et al. (2019). This further highlights the need for a more immersive memory palace experience for better recall results, perhaps starting with using a frictionless platform or omnidirectional treadmill instead of motion sensors for more natural navigation that does not leave participants thinking about their position in the real world while in the virtual one.

All participants except one agreed that they would use the VR MoL environment system as a way to memorize things in a real-life situation. Participant P3 said that it was fun and slightly less stressful compared to traditional memorization. Participant P1, who used a MoL strategy that did not take into account the order of the items, believed it would be effective as long as the memorized information did not need to be ordered. Some participants agreed with this sentiment, while others believed it was effective for ordered memorization. Those who thought it was not as effective for ordered memorization did tend to have worse strict scores compared to those who thought it would be effective for ordered memorization. Participant P7 believed it would be more effective if they had a more familiar environment to use, such as a simulated version of their real home, or just being given more time to get comfortable with the current environment. Only participant P9, who had 100% on their lenient score for both pre-test and post-test in week one when they did the questionnaire, stated that they would not use the technique, saying that they did not think it was practical to use over traditional techniques. However, in the second week, P9 recalled 10% more with VR MoL compared to their traditional studying technique.

## Conclusion

### Summary

In this study, a new virtual memory palace simulation was developed for use of the Method of Loci mnemonic technique, using virtual reality technology. This simulation took into account numerous suggestions, predictions, and past results from other studies of virtual memory palaces and improved upon the design. With the newly designed memory palace, a group of participants tested whether they could use the system to memorize a list of words more effectively than traditional studying techniques. It was found that participants on average seemed to be able to remember approximately 20.4% more words when using the virtual memory palace for the first time, and various insights were observed that could potentially further boost memory recall improvements in future VR MoL studies. When participants used the system a second time they appeared to remember approximately 22.2% more compared to traditional studying techniques. With such encouraging results, it is hoped that further studies will be conducted in the area of VR MoL, perhaps leading to further encouragement for people to make use of a VR MoL tool in their daily lives for general memorization with the help of experiments involving a greater number of participants. There is great potential for this technology especially among students studying new topics and those whose memory recall has worsened over time. We believe that this study shows that VR MoL holds a potential that applies to everyone, offering the chance for a boost in memory recall for even those not particularly in need of it, or at the very least, the ability to feasibly learn and use the complicated MoL technique in a short time.

In the spirit of furthering science and this work, the following resources are provided:

- Source code for the VR MoL memory palace: https://bitbucket.org/BrighamMoll1/thesis-experiment-vr-mol/src/master/
- The full Computer Science Thesis from which this work is based: https://bitbucket.org/BrighamMoll1/thesis-experiment-vr-mol/downloads/
- Collectibles Functionality Demo (YouTube): https://www.youtube.com/watch?v = FGExhxYDQog
- Placeables Functionality Demo (YouTube): https://www.youtube.com/watch?v = MNo7H8tN0uQ

We hope this will encourage other researchers to explore and extend our work.

### Limitations

The arrival of COVID-19 led to multiple limitations in this research. Participants were selected through convenience sampling rather than random sampling, and the number of available participants was limited because COVID-19 restrictions regarding human contact was only permitted within small social circles. This inevitably led to possible bias in results due to having a small and non-random sample size. The situation also led to the inability to acquire a frictionless platform such as one of KAT VR’s KAT Walk products, leading to the inclusion of KAT VR’s KAT Loco worn motion sensors instead. Originally, we had planned for 40+ participants from Sheridan College. We understand that lacking participants has underpowered the potential conclusions of this study, but simultaneously, we also believe that the study still holds great value in showing the feasibility of VR MoL optimization through various techniques, and additionally, there is value in the VR MoL environment we built that is now open-source and available to other researchers in the field.

In our study there was no control group, as we believed teaching traditional MoL would be too time consuming to fit within our time constraints. Ideally, there should be a group doing no intervention and a group using the MoL without the virtual reality, to assess the real benefit of using the technology compared to traditional cognitive interventions (i.e., teaching memory techniques in a classroom-type setting).

Due to concerns about first-time users of VR getting nausea, as well as time constraints, the word lists that participants were tested with were limited to 30 words rather than the minimum of 40 words suggested by Ross and Lawrence (1968).

The limitations we faced in conducting this study made it one that seeks to prove the feasibility of MoL optimization through VR rather than proving it has been done here. We acknowledge that there is not enough data to make a conclusion about the true effectiveness of our VR MoL environment on participants as a whole, and we hope that other researchers with the opportunity to study more thoroughly with the environment will be able to make more robust conclusions.

### Future work

There are a number of suggestions for future work with VR MoL that can be extended from this study. Greater immersion has been observed that results in better recall, as is evident from comparisons between how well people did and their rated level of immersion in the questionnaire at the end of the experiment. This is also a trend that has been observed in other previous studies. We suggest that in a future study a frictionless platform could be used for walking around the memory palace, such as KAT VR’s KAT Walk products. Alternatively an omnidirectional treadmill could be used, but it may not feel as smooth, and may make more noise, than a truly frictionless platform, leading to less immersion for the participant; however, more research is required to confirm this. Also in the realm of studying greater immersion, it could be useful to study if there might be a relationship between higher participant presence (people feeling like they are truly in the virtual environment) and memory recall performance improvements.

Another suggestion for future studies would be to increase the length of the word lists used to test participants to at least 40 words, as suggested by Ross and Lawrence (1968), if not more than 40. A word list of 30 words was suitable for our research; however, increasing this size challenge for some participants to clearly see the improvements in memory recall due to using the Method of Loci versus traditional studying techniques. This was not done in this thesis due to time constraints and concerns about feelings of nausea in participants, but with the combination of a more immersive walking method such as a frictionless platform it is believed that longer word lists could be used without issue as long as time permits. In this experiment, 30 seconds per word to be memorized seemed like a comfortable amount of time for participants to complete the VR MoL studying phase of the experiment, but the time required for studying may need to be investigated further.

Further studies could also investigate the effects on recall performance by providing participants more training time with our VR MoL palace (e.g., weeks or months). We believe that with more chances to explore the system and get familiar with both the VR equipment and the simulated apartment palace, participants could improve on their recall scores. Throughout the extended training period, participants could also be guided with a more detailed description of the MoL technique from the start of the VR MoL phase that outlines how they should look for locations that seem to memorably match their object in their nearby vicinity rather than just place the object in the best perceived location within the whole palace. Participants would be guided to use a well-defined, non-overlapping path through the rooms that they choose for themselves.

Another area along this train of thought is to include traditional neuropsychological tests of memory with population norms to measure if there’s a correlation between the performance in virtual reality and standardized pen and paper cognitive tasks. Such a study may further reveal the potential impact VR has in the context of improving memory recall.

Another enhancement that further supports the MoL method is to strengthen the linkage between objects in the virtual environment by imposing an order on the words as they are presented. Future work could explore adding a sequence number on each*Placeable* as they are placed (i.e., ‘1,’ ‘2,’ etc.).

It may also prove useful to study how well someone can remember words from the first time they used the system, several minutes, days, or weeks later, to investigate the power of VR MoL, in terms of long-term memory. Numerous studies have already shown the usefulness of the MoL for long-term memory recall, but perhaps with further refinement of the virtual memory palace simulation, improvements to long-term memory could be increased far more than with a traditional MoL approach.

## A. Appendix

### A.1 Questionnaire

Table 9 presents the survey questionnaire that was administered for the qualitative investigation portion of the study.

**Table 9:** Questionnaire

1.	What strategy did you use during the first memory recall test to memorize the words shown to you before entering virtual reality?
2.	How much experience did you have in virtual reality prior to this experiment? (Rating your experience from 1 (not at all experienced) to 5 (very experienced))
3.	Did you use the Method of Loci (MoL) in the second memorization test, after using virtual reality, as instructed?
4.	How confident were you with your answers in the first recall test (before VR)? (Rate your confidence from 1 (not confident at all) to 5 (very confident))
5.	How confident were you with your answers in the final recall test (after VR)? (Rate your confidence from 1 (not confident at all) to 5 (very confident))
6.	How immersive did you find this environment? Or, how much did you feel you were *in* the virtual world? (Rate from 1 (not immersed) to 5 (very immersed))
7.	Before the experiment, did you have any knowledge of what the Method of Loci (MoL) was?
8.	If you had the chance to use a system like this to memorize things in a real-life situation, would you? Why, or why not?

### A.2 Experiment protocol (Script)

Table 10 presents the protocol that was used by the researchers in conducting the main experiment.

**Table 10:** Experiment protocol

1.	Request participant to do five training mental rotation tasks, and ten testing rotation tasks. Record their results into a spreadsheet
2.	Give 15 minutes (max) for participant to study a list of 30 words and memorize as many as possible, in order if possible. (Pre-test word list print off)
3.	Give 5 minutes (max) for participant to write down every word from the list, in order if possible, in another room. (Pre-test testing paper print off)
4.	Explain the ‘Method of Loci’ using the following description: ‘In this method, memory is established from places and images. If we wish to remember an object, we must first imagine that object as an image, and then place it in a location. If we wish to remember a list of objects, then we must make a path out the many locations. The easiest way would be to imagine a familiar environment and place the imagined objects inside it. Then, you can pick up the objects as you imagine navigating the environment, thereby remembering the object list in order’
5.	Ask if the participant has any questions about the technique or if they understand. Answer any questions about how it works. Instruct them that they will be using the technique in the post-test
6.	Give roughly 10 minutes for the participant to get into the VR equipment and explore the environment. At this point, brief the participant on what controls there are on the VR controllers, and which buttons do what in the VR environment. This way when they put the headset on, they won’t be searching for the buttons too much. Once within the simulation, show them how to show and hide the progress display. Instruct them to stand somewhere in the center of the play space available to them in the real world, and try to correct their position whenever they get too close to a real wall. Have them walk around physically using the worn sensors, and collect each of the collectibles in the environment as a way to explore it (Press ‘1’ on the keyboard to initiate this phase.), using their hands without pressing any buttons. Use the training object list (Press ‘2’ on the keyboard to initiate this phase.) to have them practice selecting and placing 5 Placeables before the real test. Ensure the participant knows they are not meant to memorize these training words. Let them explore if they feel the need to (within the 10 minute time limit), until they are ready for the final test. If they need more than 10 minutes for this, allow it if possible
7.	In the test, have them place several Placeables and try to use the Method of Loci to remember all the items they place, and in what order they were placed. Tell the participant to try to place each item in a place in the palace that you will remember well as matching with the item. Also, make sure you pick pictures for each item that match what you think would be most memorable to you. To remember the order of the items, you can try to remember the path you took through the palace when you placed each item. If you have any spare time after placing all the items during the test, you can use that time to explore the environment and try to remember where you placed everything”
8.	When they are ready, begin the Placeables (main experiment) phase. Allow the participant to place every object until there are none left, within the 15 minute time limit. Afterward, if they have spare time, they can explore and try to remember where everything is
9.	When finished, press ‘9’ on the keyboard to save the participant’s results
10.	Next, send the participant back to the testing room, where they will write down as many of the 30 items as they can, and if possible, in the order from the palace, using the Method of Loci. They have 5 minutes to do this, maximum, as in the pre-test
11.	Finally, have the participant answer the questionnaire for the experiment

### A.3 VR controls

Table 11 presents the controls on the VR Controllers to perform actions in the memory palace.

**Table 11:** VR controls

Controller Button	Description
‘A’ Button	Show progress menu
‘Y’ Button	Confirm Placeable placement. Will freeze the Placeable in place and generate the next image selector if there is another word to place in the word list.
‘X’ Button	Return Placeable to participant (So that if a glitch happens or the Placeable gets thrown somewhere it can quickly be brought back
Middle-finger(trigger/grip) Button	Press these buttons and put the correct hand next to a Placeable to grab it, release button to drop the item.

### A.4 Keyboard controls

Table 12 presents the keyboard controls for the experiment.

**Table 12:** Keyboard controls

Key	Description
1	Collectibles phase
2	Training Placeable phase
3	Testing Placeable phase
9	Save participant data
0	End current experiment phase
